# Health workers’ experiences with digital and cash payments during health campaigns in Nigeria: a qualitative study

**DOI:** 10.3389/fdgth.2026.1780807

**Published:** 2026-10-23

**Authors:** Jesuloluwa Alabi, Folashayo P. Adeniji, David A. Adewole, Segun Bello, Juliet Aweko, Elizabeth Ekirapa, Peter Waiswa, Olufunmilayo I. Fawole

**Affiliations:** 1 Department of Epidemiology and Medical Statistics, Faculty of Public Health, College of Medicine, University of Ibadan, Ibadan, Nigeria; 2 Department of Health Policy and Management, Faculty of Public Health, College of Medicine, University of Ibadan, Ibadan, Nigeria; 3 Department of Health Policy and Behavioral Sciences, School of Public Health, Georgia State University, Atlanta, GA, United States; 4 Department of Health Planning, Policy and Management, Makerere University School of Public Health, Kampala, Uganda

**Keywords:** digital payment, health campaigns, health workers, motivation and performance, Nigeria

## Abstract

**Background:**

Digital financial services (DFS) for health worker remuneration have introduced a paradigm shift, addressing many of the challenges associated with cash payments. However, evidence on health workers’ experiences of digital and cash payments in Nigeria remains limited. This study explored health workers’ experiences and perceptions of digital and cash payments, with particular attention to satisfaction, motivation, perceived work performance, and the benefits and challenges associated with each payment modality.

**Methods:**

This qualitative study was conducted among 171 purposively selected immunization campaign workers from three regions of Nigeria, including the Federal Capital Territory. Participants included both operational and strategic-level workers, such as local and state immunization officers. Data were collected through in-depth interviews (*n* = 12), key informant interviews (*n* = 12), and focus group discussions (*n* = 12; 147 discussants). Data collection was conducted from May to July 2023, and each interview lasted about 35 min. All interviews were audio-recorded, transcribed verbatim, coded inductively, and analyzed thematically. The National Health Research Ethics Committee of Nigeria (NHREC/01/01/2007) approved the study.

**Results:**

Campaign workers generally perceived digital payments as supporting their satisfaction, motivation, and willingness to perform assigned campaign activities compared with cash payments. Digital payments were valued for their transparency, direct funds transfer, timeliness, reduced intermediary involvement, and lower travel-related costs. Participants also described challenges, including delayed payments due to incorrect account information, difficulty resolving transaction problems, limited access to smartphones or bank accounts, and unstable internet connectivity, particularly in rural areas. Some participants therefore continued to prefer cash because it provided more immediate access to funds and depended less on digital infrastructure.

**Conclusion:**

Digital payments were beneficial when remuneration was timely, accurate, and transparent, but depended on reliable implementation and infrastructure. Strengthening account verification, providing timely mechanisms for resolving payment problems, improving user training, and ensuring reliable connectivity may improve payment experiences and support health workers’ motivation and perceived work performance.

## Background

The twenty-first-century digital technology has revolutionised the way of life in virtually all spheres of human endeavour, and the economic impact can be compared to the gains of the nineteenth-century industrial revolution (1). Digital financial services (DFS) have piggybacked on the availability of global internet services and the data systems’ interoperability to facilitate operations such as banking, insurance, bill payments, and mobile transfers (2). The traditional use of cash payments in health programmes has been linked to significant challenges, including operational inefficiencies, poor documentation, delayed or incomplete disbursements, and security risks (3). Most organisations that engage health workers (3), have transitioned from cash to digital payments because of greater transparency, donor confidence, and financial accountability.

Electronic payment systems offer ample opportunities to improve the overall performance of the health system, due to their relative ease of use and payment efficiency. These new technologies provide more straightforward and quicker methods for conducting financial transactions than the use of cash for payments (3). Digital financial services (DFS) are increasingly integrated into health programme delivery and may support more efficient implementation of programme activities and achievement of programme goals (1). Hence, DFS offers prospects for global health initiatives and programmes to advance more rapidly towards their objectives and desired outcomes.

However, the potential benefits of digital payments may not be fully realized in resource constrained settings, where financial and digital exclusion remain important barriers to implementation (4). Previous research has identified challenges associated with digital payment deployment, including limited network coverage, weak personal identification systems, cyber fraud, transaction costs, and difficulties accessing digital financial services (4). The efficient and timely payment of health workers may also be constrained by the large number of informal health workers, inadequate electronic and internet infrastructure in remote areas, and limited financial literacy, particularly among lower cadre health workers (5). These challenges may affect workers’ ability to access and manage their remuneration even after payment systems have transitioned from cash to digital platforms.

These issues are particularly relevant to health programmes that rely on large numbers of workers deployed across geographically dispersed settings, including immunisation campaigns. Although digital payment systems may offer advantages in transparency, accountability, and efficiency, their implementation may also introduce challenges related to account verification, connectivity, access to funds, transaction management, travel requirements, and digital literacy. These experiences can provide insight into how the transition from cash to digital remuneration is experienced in practice, including the factors that may facilitate or constrain its implementation.

Evidence on the influence of digital payment systems on health workers’ satisfaction, and performance in resource-constrained settings such as Nigeria remains limited (6). The existing literature suggests that digital payment systems may improve the efficiency, transparency, and accountability of health programme payments, while also highlighting practical and contextual challenges that can affect how payments are accessed and managed (3, 6). However, much of this evidence has focused on the operational or system-level benefits and implementation barriers of digital financial services, with comparatively less attention to how health workers themselves experience different payment modalities and how these experiences relate to satisfaction, motivation, and perceived work performance. There remains limited qualitative evidence from resource-constrained settings such as Nigeria examining the processes and contextual conditions that shape these experiences.

This study is part of a larger study that quantitatively and qualitatively assessed the health campaign workers’ experiences with transitioning from cash to digital payment method of renumeration and how this relates to work related outcomes including motivation, satisfaction and performance. The quantitative study examined associations between digital payment arrangements and selected health worker outcomes, whereas this qualitative study examines how health workers and health system stakeholders experienced and interpreted the transition from cash to digital payments. In particular, the qualitative data provides insight into the mechanisms and contextual conditions that may explain perceived differences between payment modalities, including payment timeliness, transparency, intermediary involvement, account verification, access to funds, travel costs, financial management, and digital and infrastructural barriers. The qualitative findings therefore contribute to understanding how and under what circumstances different payment modalities may shape health workers’ experiences, motivation, and perceived work performance.

### Conceptual framework

Figure 1 presents the conceptual framework developed from qualitative themes emerging from the study. The framework positions payment modality (digital or cash) as the starting point, followed by payment process features, including timeliness, accuracy, transparency, and accessibility, which shape health workers’ experiences of convenience, trust, stress, and payment related problems. These experiences are conceptually linked to satisfaction and motivation, reflected in satisfaction, confidence, willingness, and commitment, and subsequently to perceived work performance, including effort, participation, and task completion. Contextual and implementation factors, including access, connectivity, verification processes, training, and support, are represented as cross cutting influences that may shape worker experiences, satisfaction and motivation, and perceived work performance. The framework reflects inductive qualitative themes and perceived pathways identified in the study rather than prespecified relationships.

**Figure 1:**
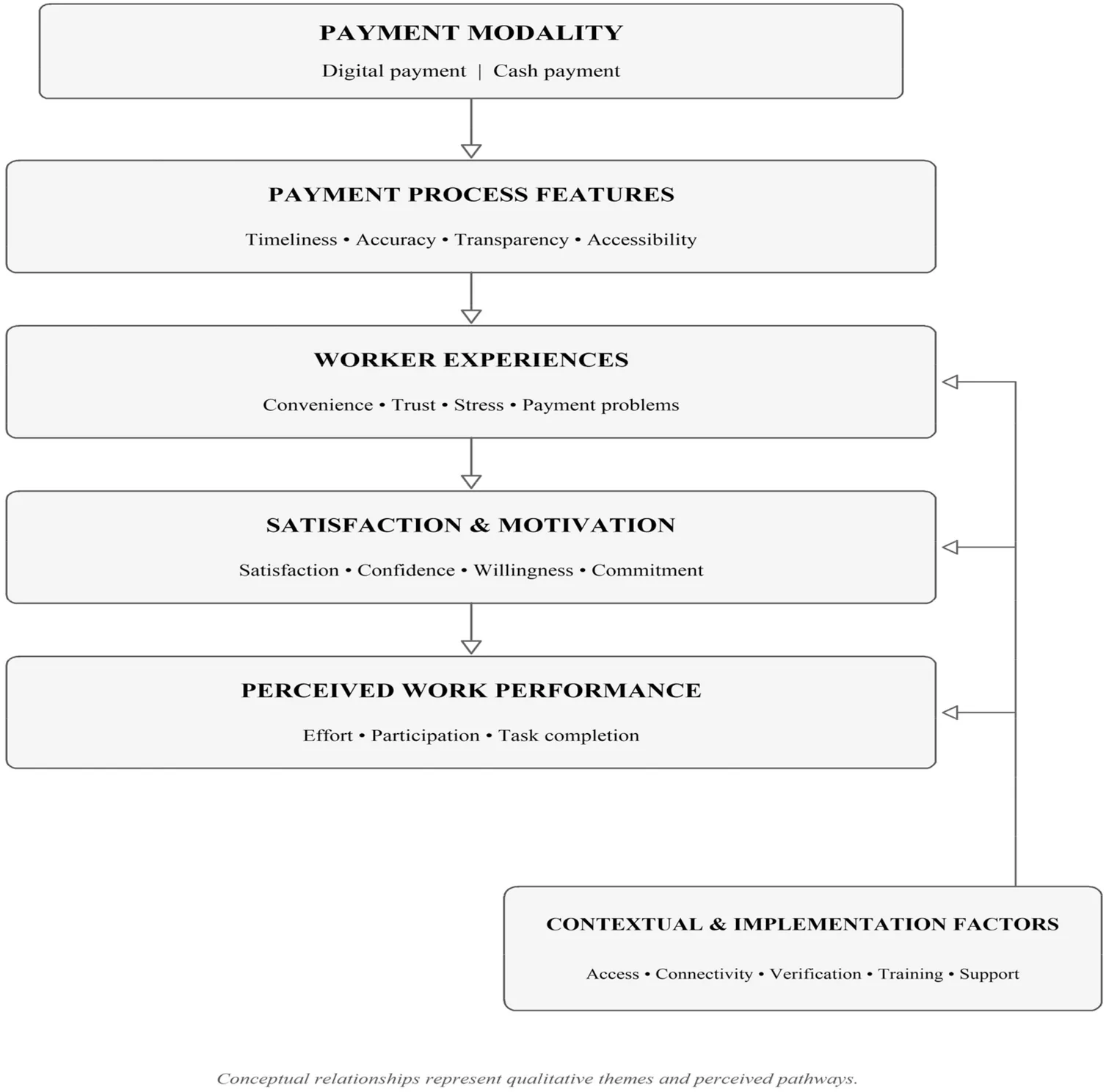
Conceptual framework for health workers’ experiences with digital and cash payments.

## Methods

### Study settings

Nigeria is divided into six geopolitical zones and comprises 36 states. In 2023, it had a total population of about 227 million people (7). More than 60 million adults live in rural areas, while about 35 million reside in urban areas (7). About half and a quarter of the rural and urban populations, respectively, are financially excluded (8).

### Study design and approach

This qualitative study was conducted as part of a broader mixed methods research project examining digital payments among health workers involved in immunisation campaigns in Nigeria. The present manuscript reports exclusively the qualitative component of the study, comprising in depth interviews (IDIs, *n* = 12), key informant interviews (KIIs, *n* = 12), and 12 focus group discussions (FGDs) involving 147 discussants. The qualitative component was designed to explore health workers’ and health system stakeholders’ experiences and perceptions of digital and cash payments, including perceived effects on satisfaction, motivation, and work performance, as well as the benefits, challenges, and contextual factors influencing the use of each payment modality. Quantitative findings from the broader project have been reported separately (6).

### Study population

To obtain comprehensive information, the study population comprised two broad groups: health workers at the strategic and tactical level of the health system and health workers at the operational level. Health workers at the strategic and tactical level included the State Immunisation Officers (SIOs) and Local Immunisation Officers (LIOs). These participants took part in the KIIs and had long-term experience in organizing and supervising immunisation activities and payments. Health workers at the operational level, including Community Health Officers (CHOs), Community Health Extension Workers (CHEWs), and Junior Community Health Extension Workers (JCHEWs) who were involved in campaigns such as polio and measles immunisation, participated in the IDIs and FGDs. All participants had experience with cash and digital payments.

### Sampling

Purposive sampling was used to identify participants for the IDIs, KIIs, and FGDs. Three participants were selected per state across the three regions (East, North, and West) of Nigeria, including the Federal Capital Territory (FCT) (total = 12) for both the IDIs and KIIs. In addition, three FGDs, each comprising six to eight participants, were conducted in each region, including the FCT. The FGDs considered gender and the rural-urban distribution of participants. The number of interviews and focus group discussions was determined using an iterative approach to data adequacy. Data collection continued until additional interviews and focus group discussions were no longer generating substantively new codes or themes relevant to the study objectives. A total of 147 discussants participated in the FGDs; hence, the overall number of qualitative participants was 171.

### Definition of key concepts

In this study, performance refers to participants’ perceived ability and willingness to carry out their assigned campaign responsibilities, rather than objectively measured indicators such as vaccination coverage, absenteeism, or reporting completeness. Participants’ descriptions of performance included willingness to undertake assigned tasks, work harder, travel to hard to reach areas, and continue participating in campaign activities. Motivation refers to participants’ perceived willingness, commitment, and enthusiasm to perform their assigned campaign responsibilities. Digital payment refers to the electronic transfer of campaign remuneration to workers through digital financial channels, including bank accounts and mobile money.

### Study instruments and data collection

Data were collected using semi-structured interview guides developed for each participant category. The IDI interview guide collected information on participants’ experiences and perceptions of digital and cash payments, including perceived risks, unintended consequences, and the influence of digital payments on satisfaction, motivation, and performance. The KII interview guide explored stakeholders’ perspectives on leveraging digital payments to improve the performance of community health workers and outreach frontline health workers. The FGD guides explored similar thematic areas as the IDIs but also allowed the discussant to share and reflect on the collective experiences with DFS. The study research team developed the interview and FGD guides based on the study objectives and relevant literature on digital financial services and health worker remuneration. The research team reviewed the guides for content relevance, clarity, and alignment with the study objectives. They were pretested with health workers selected from primary health centres in another state to assess the clarity, sequencing, and appropriateness of the questions. Feedback from the pretesting informed minor revisions to the wording, sequencing, and probes before subsequent data collection. All guides contained well-structured probes, were pretested for clarity and relevance, and were adjusted to ensure cultural appropriateness and alignment with study objectives. Data was collected between May and July, 2023. Methodological triangulation was ensured by combining data across FGDs, IDIs, and KIIs, with attention to both convergent and divergent perspectives.

### Data management

All interviews were audio-recorded with participants’ permission. The audios were transcribed verbatim, and an inductive coding approach was used in NVivo. Emerging themes and subthemes were identified, categorized, and reported quotes. Respondents’ quotes of their perceptions on the benefits and drawbacks of cash and digital payments on health workers’ satisfaction and motivation quotes were coded on a range of one to four pluses (+), to indicate the number of respondents who gave similar responses on a particular sub-theme. A single plus (+) sign showed that a few respondents mentioned the theme, while two pluses (++) showed that some respondents noted it. Three pluses (+++) indicated that many respondents discussed the theme, and four pluses (++++) signified that a theme was expressed by the majority or all of the respondents (9). To enhance coding consistency, members of the research team independently reviewed and coded a subset of transcripts using the developing coding framework. The research team discussed coding differences and refined the coding framework through consensus before applying it to the remaining transcripts. The team used discussions to review emerging codes, resolve discrepancies, and ensure themes remained grounded in the data. Given the inductive and interpretive nature of the thematic analysis, the team established coding agreement through discussion and consensus rather than quantifying it with an intercoder reliability statistic.

### Reflexivity

The research team recognised that researchers’ institutional affiliations, professional experiences, and involvement in work concerning health systems and digital financial services could influence the questions asked, interactions with participants, and interpretation of the findings. To minimise this potential influence, the interview guides included questions about both the benefits and challenges of digital and cash payments, and the analysis considered positive, negative, and context dependent experiences reported by participants. The research team discussed emerging interpretations during the analysis process and retained participants’ accounts of barriers and adverse experiences alongside perceived benefits. The findings are therefore presented as participants’ reported experiences and perceptions rather than as an assumption that digital payments are inherently superior to cash payments.

### Ethics approval and consent to participate

Ethical approval was obtained from the National Health Research Ethics Committee of Nigeria (NHREC/01/01/2007). The project followed the moral principles for medical research involving human subjects, as outlined in the World Medical Association Declaration of Helsinki (10, 11). The study aims and objectives were explained to participants, after which written informed consent was obtained before proceeding with the interviews. Participation was voluntary, and respondents were assured that their information would be kept confidential. The study did not collect personally identifying information, such as names or addresses. Respondents were informed of the average interview length. Data were saved on a password-protected project computer, and access was limited to designated investigators.

## Results

The characteristics of the study participants are presented in Table 1. FGD discussants were predominantly female (73.5%), while IDI and KII participants were predominantly male (58.3% and 91.7%, respectively). Participants had substantial work experience, ranging from 2 to 14 years among FGD discussants, 7 to 17 years among IDI participants, and 16 to 34 years among KII participants.

**Table 1:** Sociodemographic characteristics of study participants.

Characteristic	FGD discussants (*n* = 147)	IDI participants (*n* = 12)	KII participants (*n* = 12)
Age range, years	36–55	36–55	41–59
Sex, *n* (%)			
Female	108 (73.5%)	5 (41.7%)	1 (8.3%)
Male	39 (26.5%)	7 (58.3%)	11 (91.7%)
Location, *n* (%)			
Rural	78 (53.1%)	4 (33.3%)	4 (33.3%)
Urban	69 (46.9%)	8 (66.7%)	8 (66.7%)
Years of work experience	2–14	7–17	16–34
Health worker cadre/role	CHO, CHEW, JCHEW	CHO, CHEW, JCHEW	SIO, LIO

FGD and IDI participants were operational-level health workers, including Community Health Officers (CHOs), Community Health Extension Workers (CHEWs), and Junior Community Health Extension Workers (JCHEWs). KII participants were primarily strategic- and tactical-level health workers, including State Immunisation Officers (SIOs) and Local Immunisation Officers (LIOs).

### Comparison of cash and digital payment in relation to health workers’ satisfaction and motivation

As shown in Table 2, respondents/quotes reported more benefits and more drawbacks of the digital system than of cash payment. For example, digital payments were considered more beneficial because they were transparent and timely (+++), there was no need for intermediaries (++), payments were received directly (++), and a savings culture was encouraged (++++), while cash was considered more advantageous because it enabled easy access to funds. Regarding the drawbacks of the digital system compared to cash payments, more respondents had difficulties resolving bank challenges (++++), needed additional resources to retrain campaign workers (+++), and had discrepancies in account details (+++), sometimes due to the submission of other people’s details.

**Table 2:** Health workers’ satisfaction and motivation by type of payment: cash vs. digital.

Theme	Sub-theme	Cash	Digital
Benefits	Transparency	+	+++
	Timely	+	+++
	Payment received directly, no need for an intermediary	+	++
	Encourages a saving culture	+	++++
	Assurance of full payment	+	+++
	Reduced travel-related costs	+	+++
	Minimal fraud	+	+++
	Reduced bureaucracy/bottlenecks	+	+++
	Easy access to funds paid	+++	+
Drawbacks	Payment is centralized; resolution of bank challenges is difficult	+	++++
	Discrepancies in account details	-	+++
	Stress (time and transport)	+++	+
	Insecure/ safety	+++	+

### Benefits of cash payments

Table 3 presents the benefits of cash payments as stated by respondents. Cash payment was considered beneficial because it was timely and ensured health workers would be paid. The respondents stated that with cash, money was often received before campaign activities commenced. Workers also mentioned the absence of technical failures and the ease with which they track failed transactions when they occur. Also, cash payments were stated to enhance job performance, since transport allowances were given at the time of payment, which eased travel for fieldwork.

**Table 3:** Health workers’ experiences of the benefits of cash payments.

Themes	Emerging subthemes	Examples of quotes
Satisfaction	Timeliness of payment	…. before, the money was always sent before we started the work. Everyone would have gotten his or her money a day before when it was by cash [Participant 2, CHO, FCT].
		I prefer cash payment, because it gets to me on time without delay [Discussant 11, JCHEW, FGD Oyo 1].
		…. because cash payment doesn’t take time, that’s the number one reason it doesn’t take much of our time [ Discussant 7, CHO, FCT IDI].
		…. When it was cash payment, within a week everyone came to get their money, but with this digital method, they never got their money after one month or two months, so it was a problem for them [Participant 2, CHO, FCT].
	Timeliness in notification of failed transaction	I will prefer the cash, because if there is any issue, you can lay your complaint, unlike this account of a thing, when you come, they will tell you go to your bank and complain, but with cash payment, you will know where the problem is coming from and you will know who to channel the issue to [Discussant 3, CHEW, FCT FGD].
	Certainty of payment	For me, I would prefer payment by cash over the bank account payment, because at times, after the money has been sent to the worker’s bank account, we don't see the alert. [Discussant 3, CHO, FCT FGD].
		… but when they paid cash, everybody got their money. You identify yourself with your card and passport, and they pay you [Participant 5…, CHO, FCT KII].
		Before now, they usually did table payments (cash), which was fantastic. Everybody who works will get their money [Participant 6, CHO, Oyo KII].
		As for me, cash payment is the most preferable, because when it is payment time, you go with your ID card, they will see you and give you your money, but if it is E-payment, at times they do ghost workers (paying fictional persons), because they don’t see the person to pay. But as for cash payment, you will see my picture, my name, and all my details in the record, and I will be the one to collect the money [Discussant 10, CHO, Sokoto 2 FGD].
	Reduced technical failures	……… but with cash payment, healthcare workers don’t have the problem of reconciling their bank details to receive their payment, [Discussant …, LIO, Oyo IDI].
		……. with cash payment, we don’t experience poor network, or issues of money (internet) hanging [Discussant 3, CHEW, Oyo FGD].
Motivation Performance	Transport support	If you ask them, they will say they prefer cash payment, because even though they come physically to be paid, they are given transportation, so they don’t feel it. They are given transport money for coming, so they are happier with cash [Participant 4, SIO, FCT KII].
	Improved fieldwork	The cash payment enabled people to improve their fields because they could access their money easily and use it for transport. Digital payments slowed our work, but the cash helped us improve in the field [Discussant 2, CHO, FGD Oyo 1].

Across the three qualitative data sources, participants generally converged on the view that the main advantage of digital payment was not the technology itself but the greater predictability and transparency of remuneration. Operational workers described direct receipt of payment and reduced reliance on intermediaries, while participants in leadership and supervisory roles emphasised administrative efficiency and reduced opportunities for deductions or delays. These accounts suggest that the perceived benefits of digital payment were closely linked to trust in the payment process. However, this convergence was not universal. Some participants continued to prefer cash because digital payments could be delayed when account information was incorrect or when payment problems could not be resolved promptly. Thus, the findings indicate that the perceived advantage of digital payment depended on the reliability of the supporting systems.

### Challenges of cash payments

Respondents described the challenges of cash payments (Table 4). Respondents mentioned being stressed and physically strained from waiting long hours in queues. Respondents also cited security concerns about theft or robbery as a major challenge in handling cash. Respondents also stated high transport costs to travel to payment centres and not receiving the full amount due to them as a result of fraudulent practices by intermediaries.

**Table 4:** Health workers’ experiences of challenges with cash payments .

Themes	Emerging subthemes	Examples of quotes
Satisfaction	Stress	People were discouraged when paying in cash. Most people were not satisfied because they had queued for over 3 h at the payment centres [Discussant 4…, LIO, Oyo IDI].
	Long waiting time	When I was paid in cash, I didn’t like it because of the stress, like standing in line till they called your name, but if it’s a transfer, it goes to your phone, and you can do what you want. [Discussant 4 …, CHO, Oyo South IDI].
	Physical strain	…….and the queue, sometimes you will queue under the hot sun. Some people even collapse when they are in queue. [Discussant 4, CHO, Cross River State 2 FGD].
	Logistic challenges	We suffer with the table payment, but we can be in our room, and our payment was made [Participant X, CHO, Sokoto 1 KII].
		During the cash era, we here suffered because arranging for them to come and collect the money was work. But with the e-payment, everybody will get the money from their house [Participant X, LIO, KII FCT].
	Insecurity	There is insecurity with cash; if you get your money now, before you realize, ‘agbero or yahoo boys’ (touts) will just collect your money from you unexpectedly. [Discussant 4, CHO, Cross River 2 FGD].
		…. because of the current insecure situation and because most participants live in remote areas, which makes it difficult to reach them, especially given the problem of bandits who can hit and run at any time [Participant 3, LIO, Sokoto KII].
	Fraudulence	…. but if it is by hand, some heads(bosses) can slash the money into parts and they take some for themselves, but with the account details, this is not possible [Participant 2, LIO, Cross River KII].
		In the past, someone could collect another person’s payment, but with bank transfers, it's not possible. Fraud is now reduced because no one gets another fellow's money. [Participant 1, LIO, Sokoto IDI].

### Benefits of digital payments

As shown in Table 5, respondents described digital payments as beneficial. Respondents were motivated and satisfied with digital payments because of the timeliness and reliability of payments, ease of access to funds, reduced or absent fraudulent interference during payments, and the motivation to own a smartphone, as smartphone ownership was a criterion for receiving digital payments. Respondents also mentioned that digital payments encouraged opening a bank account, promoted saving habits/culture, and improved money security.

**Table 5:** Health workers’ experiences of the benefits of digital payments.

Themes	Emerging sub-themes	Examples of quotes
Motivation of healthcare workers	Timely	People like digital transfer because they get their money on time. Nothing delays their money except if they have a problem with their bank [Discussant 2, LIO, Oyo IDI].
		The e-payment encourages me to mobilize them to bring their children out for immunisation, I know the organization will pay immediately. [Discussant 4, CHO, Sokoto FGD].
		There is that motivation, you are sure of getting that satisfaction for what you have done. With that, you will … do another campaign when it comes. You will work and get paid, …. [Discussant 5, CHO, Cross River FGD].
Satisfaction	Reassuring/Confidence	Yes, with the digital payment campaign, health workers have confidence that they will receive their allowances when they finish their work, without any problems, and that their money will be in their accounts. [Participant 10, SIO, Sokoto 2 KII].
		It is okay with digital payments because the health workers have confidence that, after working, the money will be deposited directly into their accounts. Nobody will stop/slash the cash on the way [Participant 5, LIO, Cross River KII]
		Yes, this e-payment motivates health personnel because they know that, as soon as they present their account details and their account doesn’t have an issue, they will receive the alert immediately. Therefore, it motivates them because they know that when they work, they will be paid [Participant 10, CHO, Sokoto FGD].
	Easy accessibility	…. Easy accessibility to the money being paid to you, and it is easy to get it without any stress with no obstacles [Discussant 5, CHEW, Cross River FGD].
		The benefits are that I don’t waste my time, suffer, or get rained on. I lie down at home, the money comes in, and I keep it for my needs [Discussant 6, CHO, Sokoto FGD].
		You don’t need to travel/go somewhere to collect your money. You will be at home and still get your money [Participant 4, LIO, FCT KII].
	No fraudulent interference	It is only a few who are sincere; if some are sent to give two thousand to a sister, some of them will give one thousand and corner the one thousand. So, with digital payments, whatever you receive in your account, you will know it is for you [Discussant 3, CHO, Cross River FGD].
		I don’t know, but people don’t trust by hand. It is good that they send it directly to our account [Discussant 3, CHO, Cross River IDI].
		Nobody will touch your money, but if it is by hand, some unit heads can split it and take some for themselves [Participant 5, LIO, Cross River KII].
	Smartphone ownership	We are encouraging them to buy an Android phone. They can all operate their phones and use them to receive payments [Participant 10, LIO, Sokoto KII].
	Opening of bank accounts	It makes those without a personal bank account open one to avoid not being paid, while the rest are paid [Discussant 1, CHEW, FCT, FGD].
		Initially, those who are willing to work but don’t have an account number bring their relatives’ account numbers and names. But in our own ward here, my madam (name withheld) won’t accept. So, you must have your own account [Discussant 4, CHO, Cross River FGD].
	Improved security	If you go to another LGA to receive your payment, you may get robbed, but now they pay into the account [Discussant 7, CHO, Sokoto 2 FGD].
		Someone can rob you of the money, or you may even lose it during the cash payment time [Discussant 4, CHO, Sokoto FGD].
	Good money savings	Giving us through a bank account is more satisfactory than giving us in hand, because we save our money until we need it to buy something. Giving us cash can make us spend it however we like [Discussant 7, CHO, Sokoto FGD].
		When we were collecting cash, we must have spent more on transport to the payment centre than we collected. [Discussant 2, CHO, Cross River FGD].
		It helps me save my money, unlike when I collect cash and then go to the market to spend [Discussant 2, CHEW, Cross River FGD].
		It makes it easier for one to keep some in the bank after you remove what you want to use. If it is cash, you spend it within a few days [Participant 3, CHO, Cross River KII].
		You have to waste transport money to collect your payment in cash, but now you can be at home and receive the alert [Discussant 4, CHO, Sokoto FGD].

### Challenges of digital payments

Table 6 shows the challenges respondents encountered when using digital payments. These included irreconcilable bank details, i.e., account numbers that did not match the name on the account, resulting in delayed or failed payment. When the delay lasted for weeks or months, it turned off the timely use of funds and the ability to meet urgent personal needs. Respondents also mentioned large deductions from their bank accounts (bank charges) and poor internet connectivity, particularly in rural areas. Financial exclusion was another challenge, due to a lack of smartphones or personal bank accounts.

**Table 6:** Health workers’ experiences of the challenges of digital payments.

Themes	Emerging subthemes	Examples of quotes
Irreconcilable personal and bank details	Incorrect account number	The challenge we have is still with the incorrect account details, because no matter how much you shout, people will still give the wrong number or incomplete digits. [Participant 6, LIO, Cross River KII].
		But that bank payment sometimes, they do complain that some names are not tallying with the bank information, and that takes time to correct [Discussant 4, LIO, FCT IDI].
		Wrong account details could pose a challenge to the use of e-payment for work done by vaccinators [Participant 3, SIO, Sokoto KII].
	Using other persons’ names/ Some workers submit other persons’ account details, leading to discrepancies.	Some use other people’s names, and if another name is used, the bank will not pay such money. [Participant 1, LIO, Cross River KII].
		…borrowing the account of others, and some are not going to go and open their account. That’s why we have the issue of failed payment [Discussant 11, CHO, Sokoto FGD].
		If you borrow somebody’s account, you have to give him something. You can’t use his account just like that. [Discussant 4, CHO, Sokoto FGD].
Delay in payments		I’ve already said that most people get their cash the same day, they collect it and go away, unlike the digital, which may not be the same day. You know, banks differ, and they may alert you one or two days after- [Discussant 1, CHO, FCT, FGD].
		The payment is somewhat slow; imagine working since May and being paid 3 or 6 months later. The environment is not conducive for digital [Discussant 2, CHO, Oyo FGD].
		Delay in payment could pose some challenges to the use of epayment for work done by vaccinators [Participant 3, LIO, Sokoto KII].
Transaction failures		Most of the time, some people get the money, while others do not, even after the exercise [Discussant 4, LIO, FCT IDI].
		Failed payments are a challenge with e-payment for work done by vaccinators [Participant 3, LIO, Sokoto KII].
		Even the local chiefs complained that their people have not been paid due to failed transactions, so we should not go to any programme in their communities until we pay [Liaison Officer 2, FCT].
Poor internet connectivity		Internet failure is also a problem sometimes. The Nigerian internet is not too good for all these things, but we are managing - [Participant 1, LIO, FCT, KII]
		………. Sometimes the network hangs [Discussant 3, CHO, Cross River IDI].
Resolution of bank challenges is difficult		If you don’t receive your alert, you have to do a bank statement and go here and there before you can get your money [Discussant 4, CHEW, Sokoto FGD].
		Sometimes they (the bank) withdraw your money for nothing. You have to go to the bank to complain before they credit you again [Discussant 4, CHEW, Sokoto FGD].
Lack of ownership of a smartphone or a bank account		We tell our mobilizers from the rural areas, during training, we used to say those who did not have a phone or an account number (bank account), don’t do this training and don’t do this work. But still, they will do and they will work [Participant 9, LIO, Oyo KII].
Error in the collection and collation of bank details		They made mistakes when they came to collect our bank details. [Discussant 6, CHO, Sokoto FGD].
Incomplete payment		All…100%..of the people who gather here for payment will get their money back when they pay in cash, unlike e-payment, which may pay just 90% [Participant 1, LIO, FCT, KII].
		Digital payment delays and the mood of payments do not encourage you to work as a team supervisor, while some people under you will get paid, others will not [Discussant…, LIO, FCT IDI].
Bank charges		…. There are no obstacles apart from some charges that have been imposed by the banks [Discussant 5, CHO, Cross River FGD].
Limited accessibility in rural areas		Many of us in rural areas are participating in these programmes. I know we are in the digital world, and most of them don't have account details [Discussant 3, CHO, FCT FGD].

### Recommendations for the implementation and expansion of digital payments

In Table 7, respondents provided recommendations that may improve digital payment systems. These recommendations were: verification of the health workers’ account details before processing payments, and introduction of an app that can automatically validate submitted bank information. Respondents also recommended easy and no-cost bank account opening, particularly for health workers in rural areas. Improvement in internet connectivity, provision of smartphones, training of healthcare workers on the use of digital platforms, and prompt payment were also recommended.

**Table 7:** Recommendations for the use of digital payments .

Themes	Examples of quotes
Verification of account details	Before taking anybody’s account, the focal person must verify to avoid missed payment [Discussant 6, CHO, Sokoto FGD].
	I suggest we create an app to check the account details submitted by all participants and confirm whether they are correct [Participant 2, LIO, Cross River KII].
	If there is an application that can collate and validate each person’s account, for example, the COMCARE, if it can be improved upon or sent to supervisors to cross-check before they make the payments [Discussant 1, CHO, Oyo FGD].
Confirmation of payments from recipients	Our focal person should call us to ask if we have received the payment alert whenever money is sent [Discussant 2, CHO, Sokoto FGD].
Ease with opening accounts.	…….to continue opening accounts free for us [Discussant 10, CHO, Sokoto FGD].
Provision of smartphones and a good network	All health workers should have better phones and networks so the transfer can reach everybody [Discussant 3, LIO, Cross River IDI].
	They should improve the network. … if there is any other means to make the network easy, they can install it [Discussant 5, CHO, Cross River 2 FGD].
Instant/ prompt payment	They should pay immediately upon completion of the work, or within 3 days, so you can use it to meet your urgent needs [Discussant 3, CHO, Oyo IDI].
	People responsible for payment should improve and make prompt payments. E.g., everyone should be paid immediately upon completion of the job [Discussant 1, CHO, Sokoto IDI].
	Policy makers at the National, State, and Local government levels should find a way to address the payment delay, so that vaccinators can be better motivated to do their work. [Participant 3, LIO, Sokoto KII].
	…………. Firstly, the organization should pay on time [Discussant 4, CHO, Sokoto 2 FGD].
Training of personnel on the operation of digital payment	Firstly, we should train personnel on how to use the e-platform because, if I recall, last year we had knowledge gaps regarding the E-payment platform [Discussant 11, CHO, Sokoto FGD].
	Train people on how to input correct account details to avoid transaction failure [Discussant 2, LIO, Sokoto 2 IDI].

## Discussion

This study explored health workers’ experiences with cash and digital payments in Nigeria, with particular attention to their reported experiences of satisfaction, motivation, and work performance. It also examined the perceived benefits and challenges of digital and cash payment modalities. The findings suggest that the perceived advantages of digital payment stemmed from the characteristics of the payment process rather than through digitisation itself. Participants valued receiving the accurate amount directly, reduced reliance on intermediaries, greater transparency, and reduced costs and risks associated with travelling to payment locations. These features were perceived to contribute to greater satisfaction and willingness to carry out campaign responsibilities. However, these benefits were conditional. Account errors, delayed resolution of payment problems, limited digital access, and inadequate familiarity with the electronic platform could substantially reduce the perceived advantages of digital payment.

Previous studies have shown that health workers’ motivation and satisfaction are closely linked to achieving the health system’s goals (12–15). The use of cash for payment to frontline health workers has been associated with undesirable experiences, including fraudulent monetary deductions by superiors (14). Unsurprisingly, these experiences negatively impacted health service delivery, resulting in poor health system performance of health care workers (14). When workers are sure that they will receive the agreed remuneration, they are more likely to be satisfied and motivated to work (16). This is unlike cash payments, which, study participants stated, tend to be delayed, lack transparency, and are liable to unauthorised deductions. These drawbacks have also been noted by other researchers (3, 5, 6).

The use of digital payments for health workers’ fees has been associated with both unexpected benefits and unintended negative consequences. One unexpected benefit was that DFS could mitigate the impact of epidemics, such as the COVID-19 pandemic, on economic activities and facilitate electronic transactions, thereby promoting the tenacity of healthcare workers in continuing to deliver healthcare services despite many challenges caused by the epidemic (17). Similarly, DFS also has a protective effect, reducing the risk of transmission of contagious diseases among the general population, including healthcare workers (18, 19). This protective effect will reduce apprehension, boost the confidence of healthcare workers, and promote the continuity of essential health services during emergencies or shocks. Thus, DFS may improve healthcare workers’ morale, and performance (3), which could in turn lead to improvements in the delivery of health services and better public health outcomes (20). Recently, Nigeria’s private sector has undertaken encouraging initiatives at both the local and international levels (21, 22). A good example was the introduction of financial technology (Fintech) services into the financial sector, which are well accepted and utilised. Hence, many conventional banks have now adopted Fintech components into their financial transactions alongside their traditional banking services (23). Fintechs and conventional banks now partner with telecommunications companies to form a trio that improves the operability of DFS. This partnership, among a growing number, enables healthy competition in the private sector, which could make financial and digital inclusion easier, more cost-effective, and more efficient (21).

The increasing adoption of cashless policies by many low- and middle-income countries (LMICs), including by the Federal Government of Nigeria (FGN) through its Central Bank, is a strong initiative that could create an enabling environment for the e-payment ecosystem (22). Recently, FGN enacted a bill to incentivize and support the e-payment systems in Nigeria (24). Following this, the mobile telecommunications companies adopted wider broadband and began expanding their reach into rural and other previously inaccessible areas (24). Also, ownership of mobile telephones is on the increase (25), thus improving the efficiency of DFS in Nigeria. An increasing number of private companies have obtained licenses to provide satellite internet services; for example, Starlink and Viasat have received approval from the Nigerian Communications Commission as part of the country’s growing space sector regulation (26). These internet providers are expected to deliver faster and lower-cost internet services to Nigerians (27).

Frontline campaign workers also reported that having personal bank accounts encouraged good financial management, as another advantage of DFS. Previous studies have shown that DFS enhanced financial accountability and transparency when used to pay polio immunisation workers (3, 28, 29). In addition to supporting psychological well-being, digital payment systems also contributed to the physical well-being of healthcare workers by enabling safer, cash-free remuneration, reducing the risks and threats associated with handling and transporting physical cash (6).. By promoting satisfaction, digital payments can reduce industrial action among healthcare workers, thereby increasing productivity and improving overall health system performance. A similar positive impact of DFS has been reported in other sub-Saharan African countries (14, 30).

Despite the many advantages of DFS, including increased transparency, reduced cash handling, and improved efficiency, some frontline healthcare workers preferred cash-based remuneration. Divergent accounts are important because they show that digital payment should not be interpreted as uniformly preferable to cash. The characteristics of participants provide important context for interpreting these divergent experiences. The study included operational-level health workers involved in campaign implementation as well as personnel in strategic and tactical immunization roles, allowing the study to capture perspectives from different levels of the health system. Participants also varied in years of work experience and were drawn from different implementation settings. The composition of the participant groups, including differences in sex across the FGD, IDI, and KII groups, further reflects the range of perspectives represented in the qualitative data. These characteristics were not intended to support quantitative comparisons between participant subgroups; rather, they provide context for understanding the range of experiences and perspectives captured in the study. For workers who have experienced reliable banking access and accurate account registration, direct electronic payment reduced intermediary involvement and improved transparency. For workers experiencing account errors, poor connectivity, or difficulty resolving failed transactions, however, the same system could introduce new barriers to timely access to remuneration. The findings therefore point to implementation reliability as an important condition shaping whether digital payment is experienced as an improvement over cash. This finding adds nuance to previous evidence emphasizing the advantages of digital payment by showing that the benefits experienced by workers depend not only on the payment modality itself but also on the reliability of the systems through which payments are delivered.

Earlier evidence suggests that the main reasons for preference for cash payments are delays in digital payments or, in some cases, complete non-payment due to errors in bank details such as incorrect account numbers or mismatched account names (6). These errors not only undermine the benefits of digital payments but can also result in financial exclusion, where workers are unable to access their earned income promptly, and digital exclusion, when they are unable to participate fully in the electronic monetary system. The persistence of these challenges underscores the critical need for robust verification and data management processes when implementing DFS, as well as complementary measures, such as training for health workers and support mechanisms, to ensure the accurate submission of account information. Addressing these barriers is essential to maximizing DFS’s potential to enhance both the financial security and overall well-being of healthcare workers.

However, with digital payments, the assurance that funds would be deposited directly into workers’ personal bank accounts without interference from others was a significant motivator. Health workers were not only willing to perform assigned tasks but also to stretch themselves to do more and to remain at work (16). Inadvertently, this creates a conducive environment for improving the health system’s performance (16). A qualitative study of primary health care workers in Nigeria found that cash payments were irregular, contributing to inefficiency and absenteeism, as staff often resorted to other income-generating activities to cope with unpredictable salaries (31). In addition, systemic financial risks, such as cash-handling fraud, have been documented as threats to both institutional integrity and the safety of personnel and resources (32). A systematic review of informal payments in sub-Saharan Africa also finds that many are made in cash, driven by low and irregular health worker salaries and weak accountability (33). These findings suggest that money-based remuneration may undermine health system performance. By contrast, shifting to digital payments could provide more secure, transparent, and consistent salary remittance, reducing the risk of fraud, improving trust, and enhancing worker motivation.

The predominance of women in the health workforce is consistent with global and national trends, with women representing a substantial majority of health and care workers (34). In Nigeria, DFS can enhance the motivation, satisfaction, and overall performance of female health workers. Evidence from another study (35) indicates that over half of female health campaign workers reported managing their bank accounts in secret, highlighting how social norms shape financial autonomy and privacy. Although not universal, such constraints can limit the full benefits of digital financial inclusion. A study in Nigeria (30) further shows that female health workers with access to reliable digital payments report higher job satisfaction and motivation, suggesting that addressing gender-specific barriers to digital and financial inclusion is crucial. There is also a need to improve access to banking services and provide reliable internet connectivity in rural areas, where women predominantly experience these constraints. Such interventions could reduce exclusion from DFS (28) and strengthen overall health system performance in Nigeria and other LMICs.

The experiences of digital payment systems may also vary according to workers’ socioeconomic characteristics, educational background, and digital literacy. Workers with limited financial resources, lower levels of education, or limited ability to operate smartphones may face greater difficulties accessing, managing, and troubleshooting digital payments. These factors may therefore influence how much campaign workers can benefit from digital remuneration, particularly where reliable connectivity, technical support, or digital financial literacy are limited. Future implementation efforts should consider these differences and provide appropriate training and support to promote equitable access to digital payments.

The findings also highlight that the transition to digital payments involves financial and operational requirements that should be considered alongside its potential benefits. Although digital payment may reduce some costs associated with cash handling, travel to payment centres, intermediary involvement, and the security risks associated with transporting cash, implementation requires investment in account verification, worker training, technical support, connectivity, and mechanisms for resolving failed transactions. Participants described the need for additional training and difficulties resolving account-related problems, suggesting that these implementation requirements can impose costs on programmes and workers. Importantly, this study did not collect sufficient data to estimate the financial cost of digital payment relative to cash. Future research should therefore assess the full cost of implementation, including platform and transaction costs, training, technical support, account verification, connectivity, and the administrative costs associated with resolving failed payments. Such analyses would help determine whether the operational efficiencies of digital payments translate into cost savings.

This study’s contribution lies in examining the processes and contextual experiences underlying the use of digital payments. While the companion quantitative analysis provides estimates of associations between digital financial services and health worker outcomes, the present qualitative analysis identifies mechanisms through which payment systems may shape those outcomes.

Participants described the importance of timely and complete payments, direct receipt of remuneration, reduced intermediary involvement, and lower costs of accessing payments. At the same time, participants identified account errors, delayed resolution of payment problems, limited access to digital infrastructure, and training needs as conditions that can weaken these benefits. These findings help explain why the effects of digital payment systems may vary across workers and implementation settings.

### Study strengths and limitations

This study provides new insights into the role of digital payments for health workers’ remuneration and addresses gaps in literature. Through qualitative exploration, it captures rich, context-specific experiences and perceptions of digital payment adoption, offering policymakers practical guidance to enhance workforce efficiency, motivation, and financial inclusion. However, the qualitative study conducted across four states in three geopolitical zones in Nigeria provides an in-depth understanding. Still, they may not be fully generalizable to all regions or contexts. In addition, reliance on self-reported data from health workers introduces potential recall and social desirability biases. Despite these limitations, the study offers a strong foundation for future research and informs policy development on digital financial inclusion in the health sector.

## Conclusion

This study suggests that digital financial services can provide a more transparent, secure, and efficient alternative to cash remuneration for health campaign workers when payment systems are reliable and accessible. Participants associate direct and timely payment with greater satisfaction, motivation, and willingness to perform assigned campaign responsibilities. However, digital payment also introduced challenges, including errors in account information, delays in resolving payment problems, limited access to digital infrastructure, and training needs. These findings indicate that the benefits of digital remuneration depend on effective implementation, including accurate account verification, accessible mechanisms for resolving payment problems, appropriate worker training, and reliable connectivity. By highlighting how specific payment process characteristics may shape health workers’ experiences, this study contributes qualitative evidence on the mechanisms and contextual conditions underlying the perceived benefits and challenges of digital remuneration. More broadly, the findings suggest that digital payment initiatives should be accompanied by implementation systems that promote reliable, equitable, and accessible payment delivery. This qualitative study examined perceptions rather than objectively measured workforce outcomes, therefore further research is needed to determine whether these perceived benefits translate into measurable improvements in health worker performance, and service delivery.

## Data Availability

The original contributions presented in the study are included in the article/Supplementary Material, further inquiries can be directed to the corresponding author.
