# Supplementary material for: Health workers’ experiences with digital and cash payments during health campaigns in Nigeria: a qualitative study
Source: Front Digit Health. 2026 Oct 23;8:1780807. doi: 10.3389/fdgth.2026.1780807 (PMC13739669; doi:10.3389/fdgth.2026.1780807)
Supplement: Supplementary file 1 [file Datasheet1.docx]

**REPORT FOR QUALITATIVE ANALYSIS RESULTS ON ASSESSMENT OF THE INFLUENCE OF DIGITAL PAYMENT ON HEALTH WORKERS’, MOTIVATION, PERFORMANCE AND RETENTION**

The report presents the thematic analysis of the ten (10) transcripts from the in-depth interviews (IDI) on digital payment systems in immunisation campaigns and reveals several themes and patterns across the interviews. The outputs from the analysis of the transcripts were categorized into themes that were used to answer the study research questions/objectives. The study findings provided answers that shed light on the multifaceted impact of digital payments in the context of immunization campaigns as it aligns with the study's core objectives.

The research identified the mechanisms through which digital payments influence immunization campaigns, as it was discovered that the gradual transition to digital payment methods had substantial implications for the overall efficiency and transparency of these campaigns. By streamlining payment processes and minimizing manual paperwork, digital payments contributed to more organized and accountable immunization efforts. The study also found evidence that showed that the adoption of digital payment methods influenced health workers' satisfaction and motivation in positive ways. It was apparent that timely and reliable compensation through digital means had a positive effect on health worker satisfaction. The convenience and predictability of digital payments enhanced their motivation to participate actively in immunization campaigns, ultimately benefiting the overall healthcare system.

The research also examined the influence of digital payments on health workers' performance and retention and discovered that the adoption of digital payment systems positively impacted health worker performance during immunization programs. By eliminating uncertainties surrounding payment, health workers could focus more on their tasks, leading to improved immunization population coverage. Additionally, the reliability of digital payments contributed to better worker retention, reducing turnover rates and ensuring a more stable workforce.

Gender and social norms were explored concerning digital financial service behaviour. While gender itself did not emerge as a significant factor affecting access to digital payment platforms, the study revealed that smartphone ownership played a vital role. This underlined the importance of addressing disparities in smartphone accessibility to ensure equitable access to digital payment systems. The research findings also identified the benefits, risks, unintended consequences, and challenges associated with the use of digital payments for health workers. It became clear that while digital payments offered advantages such as efficiency, transparency, and accountability, they also introduced challenges. Issues like payment delays and ensuring the reliability of digital payment methods required careful attention. Addressing these challenges was identified as a crucial step toward maximizing the benefits of digital payment systems for healthcare workers and immunization campaigns. Using the thematic analysis model of the presentation of key data, this data point shows that eleven (11) themes fit the four (4) study objectives, and they are all listed in the thematic summary table presented below.

**Table 1: Thematic Summary for Analysis Output on IDI Transcripts**

| 1st Objective | Mechanisms through Which Digital Payments Influence Immunization Campaigns in Africa |
| --- | --- |
| **Main Themes** | - **Evolution of Payments for Immunization Programme** - **Full-Scale Adoption Digital Payment Systems** - **Promotion of Digital Payment Systems** |
| 2nd Objective | Influence of Digital Payment on Health Workers’ Satisfaction and Motivation |
| **Main Themes** | - **Positive Influence on Workers’ Satisfaction** - **Positive Influence on Community Members' Satisfaction** |
| 3rd Objective | Influence of Digital Payment on health workers’ performance and retention |
| **Main Themes** | - **Positive Impact on Workforce Performance** |
| 4th Objective | Influence of gender social norms on digital financial service behaviour |
| **Main Theme** | - **Influence of Gender Roles in Access to Digital Payment Systems** - **Influence of Gender on Smartphone Ownership** |
| 5th Objective | Benefits/Risks/Unintended Consequences and Challenges Associated with the Use of Digital Payments for Health Workers |
|  | - **Benefits and Lessons of Digital Payment Integration** - **Challenges of Digital Payment** - **Barriers and Suggestions for Improvement** |

**Descriptions of Thematic Summary**

The first research question and objective of this study were answered by the three distinct themes presented in the thematic summary table below and the implication is that the study has discovered that the payment system has **“Evolved over the years from the cash and paper-based to digital payment system”;** **“Full-scale adoption digital payment systems”** and **“The continuous promotion of the digital payment systems”** were the three mechanisms adopted by the healthcare system decision-makers in implementing the immunization campaigns in Africa in Nigeria. These three themes were coined from the information provided by the participants in the numerous conducted in-depth interviews and the originating quotes were presented in the body of the report after the table presentation.

Similarly, themes four and five were also generated and named to match and answer the second research objective/question as the study discovered that the adoption of digital payment system has a **“Positive influence on healthcare workers' job satisfaction”** and a **“Positive influence on community members satisfaction with healthcare performance during immunisation campaign”.** The findings from the second objective also linked to the third research question and objective where the influence of digital payment systems on work performance and workers’ retention was assessed, the study found that the adoption of digital payment systems also had a **“Positive impact on the healthcare workforce performance during immunization programme.”**

The study's fourth research objective/questions assessed gender and social norms as two key factors that could influence digital financial service behaviour for healthcare programme implementation with special reference to immunization programmes and it was discovered that there was an **“Influence of gender roles in access to digital payment systems”** and it also **“Influence smartphone ownership”** that resultantly influence the usage of the digital financial service. Lastly, the study found that the adoption of digital payment systems had numerous **“Benefits and lessons”**; while also highlighting **“Systemic challenges affecting the adoption of the digital payment system”** and **“Individual-based barriers and suggestions to address the barriers”.** These were the three thematic expressions that answered the fifth research question/objectives.

**Theme 1: Evolution of Payment Methods**

The majority of the participants interviewed reported the different shifts in payment methods over time, transitioning from cash payments to digital methods such as mobile wallets and direct bank transfers. The cash payment had been abandoned but, in some places, the only mode of payment is by cash. Few of the participants agreed to receive payment through wallets. To receive money via e-wallet, each worker must have an account number and any error detected could lead to no payment. They believed that payment through direct bank transfer is faster and safer than other methods. The evolution to digital payment has not been fully established as some of the respondents revealed that sometimes they receive payment through cash and sometimes bank transfers.

*“They pay by cash and transfer but sometimes they use a wallet. But for now, they are using transfer to pay”-* **[Participant 1, Ibadan South-West IDI]**

*“They do the mobile payment, money is sent to our wallet and we can go and clear from it from any bank, we do table payment, they also pay through the bank. For the table payment, you fill out a card, attach a passport on it and submit for payment.” -* **[Participant 3, Cross River 1 IDI]**

*“The payment is very good, E-payment, there is no problem about it, they only captured us and we were paid. The digital is better, there is no problem with the digital.” -* **[Participant 5, Sokoto 2 IDI]**

*“So, before the Covid-19 pandemic, we are using cash payments to all the vaccinators and others. All the workers are getting it or paying through hand cash payment. But later, when COVID-19 came, they started using payment through e-payment. Each individual will have his/her account number. And they send his money through his account directly not indirectly.” -* **[Participant 6, Sokoto 3 IDI]**

*“Before, they used to pay us hand to hand at Abib idugbe. they started using Wallet for us for payment. But that wallet is so bad, that some people can't receive their money. So now they are using our account to pay us but not all people have this account especially the ones in the rural areas.”-* **[Participant 10, Oyo IDI]**

**Theme 2: Full-scale Adoption of the Digital Payment System**

The most recent digital payment system, often involving direct bank transfers, was generally considered efficient and less stressful compared to earlier methods. However, issues related to delayed payments were reported in some cases. some of the participants reported that payment was delayed and some workers didn’t receive payment, With respect to this issue, one of the participants noted the preference for table payment (cash) as opposed to the digital payment system.

*“yes, but I don’t think anyone has no access to digital payment. We are all collecting from transfer unless there is an extra team. because most of them don’t belong to that community”-* **[Participant 1**, **Ibadan South West IDI]**

*“E-payment, I prefer table payment because it doesn’t take time let me say if you end your campaign this week in the next one week, they will call you for your payment but if it’s E-payment it will take more than a month.”* - **[Participant 4, Kuje IDI]**

“*The current digital payment system is through a bank account. At times it operates well while at times it doesn't because some complained that they don't receive the alert.”*- [**Participant 10, Oyo IDI]**

**Theme 3: Promotion of Digital Payments**

Although digital payment has provided more security, and reduces the stress of travelling to get their payment sorted, yet, some of the participants highlighted recommendations to promote digital payment systems as they still experience network glitches. The recommendation includes the need for thorough verification of accounts before sending payments to ensure accuracy and avoid delays.

*“Because of complaints from some people that they can't see get in touch with their money, those in charge of the platforms should ensure they verify every account very well before they send anything.*”- [**Participant 10, Oyo IDI]**

“*Through the campaigns we are doing. When we come for campaigns, they already tell us during the training, that if you have an account, please write to your account. If you don't have one, go and offer it. Because no more cashless policy. Only your account, through your account, you want to pay*.”- **[Participant 6, Sokoto 3 IDI]**

*“The only information I can give you is that the Nigerian network is not good generally. The only thing that can be done is to improve connectivity*.”- [**Participant 1, Ibadan South West IDI]**

*“It can be promoted by ICT experts. Look for how to improve it. Extend to participant level for access. It will reduce ties/interference with LGA. Right now it has reduced the interference between the state team and LGA team payment. It's an improvement. State members usually alter our details for personal issues. It's solved now. It can be further improved. It's almost perfect, I can't say how it can be further improved*.”- [ **Participant 9, Sokoto 1 IDI]**

*“We try to mobilize people and tell them the importance of digital payment.*” - [**Participant 5, Sokoto 2 IDI]**

**Theme 4: Worker Satisfaction**

The participants reported that the level of satisfaction was influenced by the reliability and efficiency of payment methods. While some workers were satisfied with digital payments as a result of the ease of withdrawing money. some workers identified that their level of satisfaction is not derived from the payment methods, whether they are paid or not there is always motivation and willingness to do the job. Also, payment delays affected their motivation.

“*yes, very effective. Yes. Whenever I hear about a program, I will be happy that payment will come up after a month. I like the system of digital payment the only experience* ” - **[Participant 1, Ibadan South West IDI]**

*“Ehn, some people are satisfied with the transfer, some are satisfied with going to Ibugbe to collect their money but some people including me were not satisfied with the wallet at all.*”- [**Participant 10, Oyo IDI]**

*“I am satisfied because I have a bank account and I can collect my money easily. yes, some banks require* *OTP, and some workers do not have a smartphone to access it which has affected their participation*. *”-* [**Participant 2, Cross River 2 IDI]**

*“only the digital makes me happy. The way it makes me work better, I may save some in the account maybe that time I still have some change in my hand, I can save that one but if it's cash I will scudder it that's what I feel like.*”-[**Participant 7, Gwagwalada IDI]**

**Theme 5: Community Satisfaction**

The participants interviewed reported that the communities were perceived as satisfied with the immunization program. Positive receptions during follow-up visits and cooperation were indicators of community satisfaction. The level of satisfaction differs by community, people living in Urban areas were more welcoming and satisfied with the immunization theme, while those living in rural areas had to be settled by the immunization officers before participating in the immunization programmes.

*“Yes, some are satisfied with the health worker, but some are habitually not compliant. Anything you come with is not accepted. But some are happy, are friendly with health workers.*”- [**Participant 6, Sokoto 3 IDI]**

*“Here there are misconceptions about immunization when visiting houses except for people who are aware and welcome us and our services. We still beg people with misconceptions. 90% are well satisfied.*”- [**Participant 9, Sokoto 1 IDI]**

*“They are satisfied, some of them complain about the injectables that their kids cry a lot which also affects their jobs.”-* [**Participant 2, Cross River 2 IDI]**

*“Yes, we know that they are satisfied because anytime we revisit the same location we previously visited, they used to be very happy to receive us and we relate well with ourselves and they are always ready to be at our service*.”- [**Participant 10, Oyo IDI]**

*“The rural are not much satisfied. We always have to patronize them before we can give immunizations. I am from a rural place so I know what it takes. Initially, they don’t show interest but when the program wants to end, they start bringing their children.*”- **[Participant 1** **Ibadan South West, IDI]**

**Theme 6: Impact on Workforce**

The majority of the participants interviewed reported that the choice of payment method harmed the workforce involved in immunization campaigns. The common negative impact reported was that some of the workers often dropped out from the immunization campaign due to payment delays or difficulties accessing funds and less money which could potentially affect program implementation and workers' motivation. Few of the participants noted that the payment was good and they hadn’t recorded any drop-out.

*“No, because this issue is only a few about 5% and this immunization activity is soldiers comes and goes. If I have a better offer I can leave, it's not about payment. During the implementation of this payment, people who couldn't meet the criteria of opening an account left and we recruited another with a mass dropout in 2022-2021.”-* [**Participant 9, Sokoto 1 IDI]**

*“When someone is brought in for a job and the person did the job well but at the end cannot cash his/her money, it affects the person, you cannot call the person for another job until the error is rectified.*” - **[Participant 3, Cross River 1 IDI]**

“*Yes, they are fully participating, no one has dropped out.”-* **[Participant 5 Sokoto 2, IDI]**

“*What discourages people from transferring means of payment is that it can take up to a month for them to get paid after service. If people are getting their money after a week, then the level of their performance will increase*”- [Participant 1, **Ibadan South-West IDI]**

*“People are willing to come, because the more you show your friends, you receive them early, or even since the transport if you are doing this campaign, you see your transport through your colleagues, your supervisor in your team to receive your transport, you are very happy.”-* **[Participant 6, Sokoto 3 IDI]**

**Theme 7: Gender and Access**

The participants reported that the majority of the workers were female but gender does not significantly impact the ability to operate digital payment platforms. Male and female health workers had equal access to digital platforms for payment and most of them used smartphones, but smartphone ownership was more common among men.

*“The women have it more so that’s your experience okay did they know how to use it, has it affected them or could that be an issue in the payment modernity again ownership of phone or none.”-* [**Participant 4, Kuje IDI]**

“*It depends on your work because if you come for if you did for a vaccinator, female is more than the one who is doing maybe recorder or time announcer or something like that. It depends on your work. Both, male and female have access to digital payment, transfer, bank transfer.”*- [**Participant 8, Sokoto 3 IDI]**

*“Android. Everybody is supposed to have. Before women were not given that priority but now every woman.”*- [**Participant 7, Gwagwalada IDI]**

“*Yes, male and female campaign workers have equal access to digital payment platforms.*” - **[Participant 3, Cross River 1 IDI]**

**Theme 8: Smartphone Ownership and Gender**

The majority of the participants acknowledged they use smartphones while some do not. However, smartphone ownership did not necessarily correlate with access to or satisfaction with digital payment systems.

“*I have both a ‘torchlight’ phone and an Android phone. I use both of them. males have Android phones. They can operate Android phones than females*”- **[Participant 1, Ibadan South West IDI]**

“Yes, Smartphone everyone has equal access to phones.”- [**Participant 5, Sokoto 2 IDI]**

*“Yes, I have it. Galaxy. Samsung Galaxy. Yes, it's a smartphone. Yes, yes, yes. The situation around here, female is more handling this phone with males. Because females are handling the phones, and smartphones with males.”* - [**Participant 6 Sokoto 3, IDI]**

*“Smartphone. The female has more phones. Males have smartphones but females have more.”-*[**Participant 8 Cross River 3, IDI]**

“*Android. Men have the capacity to have and use more Android phones than women*.”- [**Participant 10, Oyo IDI]**

**Theme 9: Benefits and Lessons**

The participants reported the benefits they had enjoyed with the advent of the digital payment systems which included; ease, reduced travel-related stress, security, increased accountability, flexibility, and efficiency. They highlighted lessons learned such as the need for smoother, more reliable payment processes.

*“The benefit that I know is that we have our payment digitally. Secondly, even when there is no chance to get your money immediately, the money is there safe. Also, there is less misunderstanding among the workers.*”- **[Participant 3 Cross River 1 IDI]**

“*It has its benefit; the only benefit is if you’re sitting at home, you receive your money without somebody not even temper with the money you understand.”*- **[Particpant 4, Kuje IDI**

*“It is very efficient. The more you use digital, the easier to access the money, the information has been sent to the server and the server cannot hide anything.*”- **[Participant 5, Sokoto 2 IDI]**

*“It's flexible, accessible anywhere or anytime, no discrepancy in the amount paid, there is accountability, efficient in time of time and cost, it reduces fraud risk. You cannot question if I underpaid you before. ”*- **[Participant 9, Sokoto 1 IDI]**

*“It is stress-free, the stress of travelling down to Abib idugbe has reduced as we can receive our money at our disposal but for those that don't have an account, it's a big stress for them. I have peace and joy in my mind anytime I receive my credit alert on my account*.”- [**Participant 10, Oyo IDI]**

**Theme 10: Payment for Those with Limited Digital Access**

Some of the participants living in rural areas with limited digital access reported they had to rely on others who have active bank accounts to receive payments. Some reported that even with the limited digital access they still receive alerts on their phones. This practice could sometimes lead to disputes and challenges in cases where the owner of the bank account borrowed can deny the receiving alert

“they use someone’s account, they bear the account owner's details.”- [**Participant 2** **Cross River 2 IDI]**

*“Some borrow other people’s bank account. But, I do not think there is anyone who does not have a bank account in Sokoto.”-* **[Participant 5 Sokoto 2, IDI]**

*“We cannot work with them, no account number: no participation. Participation is only on possession of a personal account number. Before in bank transfer, people drop someone else details, but problems always arise because those people deny getting an alert.”-* **[Participant 9, Sokoto 1 IDI]**

*“This happens majorly to those in the village. Those with digital platforms ask those that have around them for help so that they can make use of their bank account in order to receive money to their ends.”* - [**Participant 10, Oyo IDI]**

**Theme 11:**  **Challenges with Payment Methods**

Participants reported the different challenges associated with different payment methods. Wallet-based payments, in particular, were criticized for their unreliability and difficulty in accessing funds. The wallet payment couldn’t be accessed properly and people who are not good at operating phones found it difficult to access their money as most of the workers were old. They also have to do a form of clearance before they receive payment through wallet Some of the participants also reported that they experienced delayed payments and issues with receiving payments with the different digital platforms. While long queue was associated with cash payment and several irregularities in the amount paid to staff conducting similar tasks.

*“Yes, there was a little delay because sometimes when you get there, there is a delay before you can clear it, the last time it happened was in 2015. Sometimes you tried to get your money and the wallet will not be working, that was the only challenge”*- **[Participant 3, Cross River 1 IDI]**

“*People were discouraged when they were paying by cash. Most people were not satisfied because they queued for over 3 hours at the centres of payment. During the wallet payment period, we still faced a lot of challenges like the system of operating phones. Some people don’t understand how to operate the phone very well”***. [Participant 1**, **Ibadan South-West IDI]**

*“but that bank payment sometimes, they do complain that the name given to some names are not tally with the bank information you understand and that take time most time some people will get the money why some people will not even get the money after the exercise*” -**[Participant 4, Kuje IDI]**

“*because of the money issue. Some cannot open up; some begin to borrow still after the program.”-* **[Participant 7, Gwagwalada IDI]**

**Theme 12: Barriers and Suggestions**

The majority of the participants reported that sometimes the payments made through the digital platforms were delayed because of network problems or transaction failure. while some participants reported no issue at all. They gave suggestions that issues relating to bad networks, wrong names or account numbers should be properly addressed. They further advise that the body in charge of reimbursement should employ capable and trained minds to input immunization workers' details correctly to prevent issues with transactions.

*“Delay in the quick delivery of money into the account of some people. And suggestions- before the people in charge of sending the money through the bank finally send the money, they should be very careful and watchful against anything that they know can confuse them in order to quickly rectify it with the local government especially when it has to do with the workers account numbers before they finally send the money.”-* **[Participant 10, Oyo IDI]**

“*Network problem. Also, the distance to get to the bank for those who stay in the villages. There is transaction failure because at times transaction may fail because of wrong data submission, poor network ...Good network, correct information should be collected from the individual.*”- [**Participant 3, Cross River 1 IDI]**

“*All health workers should have a better network so the transfer can go around everybody. Sometimes network hangs. They should do their system well so the money should not hang. They should bring more programs for us to do because we are always ready to do the work*.”- [**Participant 8, Cross River 3 IDI]**

“*Wrong bank account. There is hardly transaction failure when there is a network... Train people on how to input correct account details to avoid transaction failure.”-* [**Participant 5, Sokoto 2 IDI]**

“*No as I have said earlier they will make us open an account, if you don't open an account whatever happened after the program you don't see your money you won't blame the government because they've said it earlier open an account but we don't know what is exactly happening after some program like that people are not paid even with the account number, I don't know what is happening some are not entitled to their payment I don't know*.”- [**Participant 7, Gwagwalada IDI]**
