# Supplementary material for: Health workers’ experiences with digital and cash payments during health campaigns in Nigeria: a qualitative study
Source: Front Digit Health. 2026 Oct 23;8:1780807. doi: 10.3389/fdgth.2026.1780807 (PMC13739669; doi:10.3389/fdgth.2026.1780807)
Supplement: Supplementary file 2 [file Datasheet2.docx]

**REPORT FOR QUALITATIVE ANALYSIS RESULTS ON ASSESSMENT OF THE INFLUENCE OF DIGITAL PAYMENT ON HEALTH WORKERS’, MOTIVATION, PERFORMANCE AND RETENTION**

The report presents the thematic analysis of the ten (10) transcripts from the key-informant interviews (KII) interviews on digital payment systems (DPS) in immunisation campaigns and reveals several themes and patterns across the interviews. The outputs from the analysis of the transcripts were categorized into themes that were used to answer the study research questions/objectives. The first objective of the study focused on understanding the mechanisms through which digital payments influence immunization campaigns in Africa, and it was discovered that the transition to digital payments highlights a pivotal shift from traditional payment methods to digital solutions within immunization programs. The key aspect of the study findings reveals that the adoption of digital payments streamlined immunization campaigns, making them more efficient and transparent. This transition allowed for real-time tracking of payments, reducing the risk of fraud and errors, and ensuring that health workers and campaign staff were compensated promptly and accurately.

The second objective delved into the influence of digital payments on health workers' satisfaction and motivation and it was discovered that the adoption of DPS aids in improving the healthcare workforce's overall performance. The major talking point of the study findings in relation to this objective emerges in the form of improved job performance and enhanced motivation among healthcare workers. The research found that digital payments led to higher job satisfaction, as health workers could rely on timely and predictable compensation. This, in turn, motivated them to perform their roles more effectively during immunization campaigns. On the other hand, the findings showed that the adoption of digital payments contributed to a more positive work environment, reducing financial stress among health workers. As a result, they felt more satisfied in their roles, which not only improved their performance but also had a positive impact on the overall quality of healthcare services delivered during immunization campaigns.

The third objective that examined how digital payments influenced health workers' performance and retention showed that there was a positive impact on healthcare workforce retention. The study's germane findings indicate that the adoption of digital payment systems led to higher retention rates among health workers. The reliability of digital payments eliminated uncertainties about compensation, reducing turnover rates and ensuring a more stable and experienced workforce in immunization campaigns. One other significant aspect of the findings that also related to the same third objective is how there was also a positive impact on immunization population coverage. The study revealed that health workers' improved performance, facilitated by digital payments, led to better coverage rates in immunization campaigns. The consistent and reliable payment systems incentivized health workers to conduct outreach and vaccination activities more effectively, ultimately benefiting the broader population.

The fourth objective which explored the influence of gender and social norms on digital financial service behaviour also showed that DPS influenced community members' perception of encouragement of financial literacy. The study findings show that communities play a significant role in promoting financial literacy, especially among women. The study revealed that communities often encouraged women to embrace digital financial services, enhancing their access and participation in these systems. Additionally, the study found that community leaders played a pivotal role in shaping the financial behaviour of their constituents. Their support and advocacy for digital financial services, particularly for women, were instrumental in bridging the gender gap in access to and utilization of these services. Gender disparity was another aspect of the findings that relate to the fourth objective. While community support and leadership were influential, the study also highlighted that gender disparities still exist in digital financial service behaviour. Women faced unique challenges, such as limited smartphone ownership, which affected their access to these services.

The last objective of the study examines the benefits, risks, unintended consequences, and challenges associated with the use of digital payments for health workers and was able to present some critical challenges and concerns that the participants had with the adoption of digital payment for the implementation of immunisation in the region. The study identified challenges such as payment delays, technical issues, and concerns about data security and privacy. These challenges, if unaddressed, could undermine the effectiveness of digital payment systems. Equally important, the study provided recommendations for improvement. These recommendations aimed at addressing the identified challenges, ensuring the reliability and security of digital payment systems, and enhancing their overall efficiency. This aspect of the findings highlights the practical steps that can be taken to maximize the benefits of digital payments while mitigating risks and challenges.

**Table 2: Thematic Summary for Analysis Output on KII Transcripts**

| 1st Objective | Mechanisms through Which Digital Payments Influence Immunization Campaigns in Africa |
| --- | --- |
| **Main Themes** | - **Transition to Digital Payments on Smooth Immunization Programme** |
| 2nd Objective | Influence of Digital Payment on Health Workers’ Satisfaction and Motivation |
| **Main Themes** | - **Improvement of Healthcare Workforce Performance** - **Promotion of Job Satisfaction** |
| 3rd Objective | Influence of Digital Payment on health workers’ performance and retention |
| **Main Themes** | - **Positive Impact on Healthcare Workforce Retention** - **Positive Impact on Immunization Population Coverage** |
| 4th Objective | Influence of gender social norms on digital financial service behaviour |
| **Main Theme** | - **Community Perception on Encouragement of Financial Literacy** - **Community Leaders Impact** - **Gender Disparity** |
| 5th Objective | Benefits/Risks/Unintended Consequences and Challenges Associated with the Use of Digital Payments for Health Workers |
|  | - **Challenges and Concerns on Digital Payment** - **Recommendations for Improvement** |

**Description and Relation of the Study Findings (Themes) with the Objectives**

The study's objectives and thematic expressions revealed critical aspects of the findings, including the transition to digital payments, the positive influence on health workers' satisfaction and motivation, the impact on performance and retention, the role of communities and leaders in shaping gender dynamics, and the benefits, risks, and recommendations for the use of digital payments in healthcare. These insights offer valuable guidance for policymakers, healthcare providers, and stakeholders aiming to optimize digital payment systems in immunization campaigns.

**Theme 1: Transition to Digital Payments on Smooth Immunization Programme**

Most respondents mentioned a shift from cash payments to digital payment systems. Digital payment systems were viewed positively, reducing issues related to cash distribution and increasing transparency. Most of the respondents interviewed in this study reported their view on the transition from cash payment to digital payment systems. Most of the participants supported the use of e-payment as a way of reducing cash payment-related issues such as delayed payment, payment failure, insecurity, the positive impact of digital payment systems, and increased transparency amidst the healthcare systems.

*“4-5years back, cash payments were used, a card was given to us with an amount to be collected when the bank officers come around, then we moved to the wallet account, which was used by the LGA team and the focal persons while supervisors and others were still paid through cash. After wallet payment, we now use a bank account and everyone is paid now through bank transfer… Everything has its advantages and disadvantages, but it is good…”* **[Participant 6, Sokoto 1 KII]**

*“Before now, it was a table payment they normally do which was fantastic. Everybody that works will get his money. But for now, they are doing E transactions…. That is cash. You will come first to fill your card and how much they are supposed to pay you, and you will collect your money in cash. ”* **[Participant 2, LIO KUJE KII]**

*“Well, I like it. I appreciate it because your money will come to you directly. Nobody will touch your money but if it is by hand, some heads can slash the money into parts and they take some to themselves but these account details are okay. They pay you directly provided you give the account details correctly.”* **[Participant 5, CROSS RIVERS 2 KII]**

*“I will say that the banking system is even more preferable than all these, I mean transferring to an individual account. With these COMMCARE systems, if they can improve on that, I think it will be okay because that mismanagement of funds, is not from the local government immunization and it’s not from the participant”* **[Participant 9, OYO KII]**

*“We started with cash payment and it was done by WHO (partners), they will send bank officials to the ward and healthcare workers will be paid in cash. Any healthcare worker who is not present on that day will have to come and collect clearance from me and then take it to the bank to collect cash. Later bank payment was introduced. ”* **[Participant 7 AMAC, KII]**

**Theme 2: Improvement of Healthcare Workforce Performance**

Health workers indicated that digital payments improved their performance. Confidence in timely payment allowed them to focus on their work without concerns about compensation.

*“Yes. Now they are very happy, but before they are feeling very.... why? you have to bring their money like you did before. you tell him this system is dynamic is almost changed so you have to bear the situation and they now become and they now become to bear the situation and almost all of them they have an account.”* **[Participant 4 LIO KWARE LGA KII]**

*“Yes. I could have said the performance has improved but before you get the participants to work for you now is another thing different, because some of them don't see their money. and before you say bring the fuel transactions let's stress these people, sometimes even you that they know you if you call them to ask for their account number, they will tell you it is a wrong number because almost all these fraudulent, scammers.”* **[Participant 2, LIO KUJE KII]**

*“Yes, we have dropouts because of no/delayed payment or failed transactions. Some of them do not want to participate in the program again because they feel that they will not be paid.’* **[Participant 3, CROSS RIVERS 3 KII]**

*“It has been improved. That cash at hand was a big problem. That one where they give OTP before you can get your money was also difficult but this account details is fine. We verify at the LIO office if the account details are correct before we can send it to the state.”* **[Participant 5, CROSS RIVERS 2 KII]**

*“Yes, it (digital payment) has many impacts on the campaign health workers. they have confidence that they are going to receive their allowances when they finish their work without any problem and you have confidence that your money is inside your account. They are receiving all their allowances through their account now. Even their transportation allowance used to be paid into their account. They can meet their daily target. Yes, it (digital payment) has many impacts on the campaign health workers.”* **[Participant 10, SOKOTO 2 KII]**

**Theme 3: Promotion of Job Satisfaction**

Health workers reported increased motivation due to the reliability of digital payments. They expressed satisfaction with the ease of receiving payments directly into their bank accounts. Most of the participants interviewed in this study reported their views on the health workers' improved motivation and job satisfaction. Most of the respondents supported the use of e-payment as a payment strategy has increased or improved health workers' motivation, including job satisfaction, becoming a benefit to the beneficiary, and satisfaction of payment directly into the banks without any intermediary. All these views reported support the impact of digital payment systems on health workers as it improves their job satisfaction level as well as becoming a motivation to work more for the organizations.

*“It is of benefit for the beneficiary knowing they have received their money from the organisation without any intermediary or someone who will manoeuvre their money. It is very easy and the staff appreciate the e-payment more than the cash payment”* **[Participant 1, SOKOTO 3 KII]**

*“They are satisfied…because I’m the one dealing with them directly, they are satisfied in the sense that before they start the work, they know the amount they will receive at the end of the day. I tell them in this LGA that if anyone doesn’t meet up with the target or even exceed it, I will drop the person and pick another person to continue the work. With that alone, they will put more effort so that I don’t drop them to pick another person.”* **[Participant 3, Cross Rivers KII]**

*“Yes, they are……. We suffer using the table payment but we can be in our room and our payment is made”* **[Participant 6, Sokoto 1 KII]**

*“ Imagine we worked together the same month on the same campaign. You were paid and I wasn’t paid I will be discouraged to want to work at another time. So they have been saying that they prefer the bank officials coming back to pay them with their money enveloped and paid by cash. Since the introduction of digital means, we have been having problems getting ad-hoc staff.”* **[Participant 7, AMAC KII]**

*“Yes, they are motivated, whether they are very motivated and they show more interest than before because they know that I’ve worked and I’m expecting something, their money will drop. So, they are more motivated and they appreciate. they are more satisfied, the money goes directly into their account*” **[Participant 9, Oyo KII]**

**Theme 4: Positive Impact on Healthcare Workforce Retention**

Digital payments were seen as a factor that encouraged health workers to remain in their roles. Some mentioned that the confidence in receiving timely payments reduced attrition rates. Most of the respondents interviewed in this study reported their view on the influence of digital payment systems as being the factors that encourage health workers to retain their roles. Most of the respondents supported that the use of e-payment as a payment strategy has influenced the health workers to remain in their roles, becoming more responsible such as attitude towards work change positively and increasing participation level in the course of the training organized by the Ministry of Health. All these views reported support the influence of digital payment systems on health workers as a factor that encourages health workers.

*“Yes, they now take responsibility, they now come to training with their own bank account. They are motivated to work and they are satisfied.”***[Participant 1, SOKOTO 3 KII]**

*“Yes, we have dropouts because of no/delayed payment or failed transactions. Some of them do not want to participate in the program again because they feel that they will not be paid”* **[Participant 3, CROSS RIVERS 3 KII]**

*“it is okay with digital payment because the health workers will have confidence that after working, the money will come to their account directly. Nobody will stop/slash the money on the way.”* **[Participant 5, CROSS RIVERS 2 KII]**

*“We have been managing like that. That is why you see that there is no continuity in most of our works. If we use this person today, next time, another person will come and we have to go through the stress of training the person again and again because if the training is not done, they will be getting different results. Every time we change staffs.”* **[Participant 7, AMAC KII]**

*“because of unemployment issues in this country, even if some people are dropping, some will still say they want it. yes, and we do refresher training for the old ones to remind them of what they are supposed to know.”* **[Participant 8, *GWAGWALADA KII*]**

**Theme 5: Positive Impact on Immunization Population Coverage**

Respondents reported that communities were more satisfied with vaccination services due to the reliability of health workers receiving their allowances. Increased participation and awareness in communities were noted. Most of the participants interviewed in this study reported their view that the communities are more satisfied with the vaccination services due to the reliability of the health workers receiving their allowances. Most of the respondents supported the use of e-payment as payment strategies have increased awareness campaigns, improved the participation level of the communities, and also influenced the communities' perception of vaccination services. All these views reported support the impact of digital payment systems on the community perception.

*“The complaint we receive is not from that side, but we are receiving complaints, especially from Fulani camps or you know the situation of this country, the insecurity places we are having. The Fulani man will say " Your people have been coming but they are not coming again". We know the challenges, but when they tell us, we tell them not to worry that they are coming”* **[Participant 3, CROSS RIVERS 3 KII]**

*“It does not affect. They don’t have any problem... they are interested and participating more than before.”* **[Participant 5, CROSS RIVERS 2 KII]**

*“No, they did not complain that their children have not been vaccinated just the community leaders complaining about workers not being paid. Due to the issue of payment, most times, it becomes difficult to reach our target because work is been done haphazardly”* **[Participant 7, AMAC KII]**

**Theme 6: Community Perception on Encouragement of Financial Literacy**

Health workers were encouraged to have mobile phones and bank accounts to receive digital payments. Training and education on using digital platforms were provided. Most of the participants interviewed in this study reported their views on the health workers in the use of mobile phones and bank accounts to receive digital payments. Most of the respondents supported the use of e-payment as a payment strategy has encourages the health workers in participation of the training and education of how to use the digital platforms, encourages in the opening of bank accounts and use of mobile phones.

*“From my own side, I think it is not educating, because even the local person in the village who attended primary school must know how to use the phone.”* **[Participant 2, LIO Kuje KII]**

*“Usually, those that don’t have phones, are using the phone number of their parents or any guardian and it's more common among the female. We are encouraging them to buy a phone, even if it is not this android, even if it is this small phone, let them have a phone. Even for their work, if we want to go for supervision because they are not staying in one place, we need to call them so they are supposed to have a phone. Sokoto North is a metropolitan LGA and the majority of the people who are working, are elites. They can operate all their phones.”* **[Participant 10, SOKOTO 2 KII]**

*“The internet payment is good because during the cash own. We here suffer because arranging for them to come and collect the money is work. But for the e-payment, everybody will get the money from their house. Some people will do things that are not in line such that I will have to beg for the person. I prefer e-payment but if you ask them, they will say they want cash. Some of them think they don’t have much money to open bank accounts”* **[Participant 8, GWAGWALADA KII]**

*“You know, the people doing the immunization program, not all of them are literate. So, when we were trying, most especially our mobilizers from the rural areas, during training, we used to tell those who did not have a phone or an account number (bank account), don’t do this training and don’t this work. But still, they will do and they will work.”* **[Participant 9, OYO KII]**

**Theme 7: Community Leaders Impact**

Community leaders played a role in supporting vaccination campaigns and facilitating relationships with health workers. Most of the participants interviewed in this study reported their views on the roles and responsibilities of community leaders. Most of the respondents supported the health workers' decisions supported the vaccination campaigns and facilitated the relationship between health workers.

*“But the issue of the community telling us that these people have not been coming... I was citing an example just for the insecurity side and this is the situation on the ground but this is the strategy we are using to resolve those kinds of issues. Many did not comply but we have the resolution of that compliance. Those works for the area council in each community.”* **[Participant 2, LIO KUJE KII]**

*“Yes, very well. Even the chiefs complained that their people have not been paid, so we should not come for any program in their communities.”* **[Participant 7, AMAC KII]**

*“Moreover, we are working together with community leaders, the leaders of all communities we entered so there is not much problem.”* **[Participant 10, SOKOTO 2 KII]**

**Theme 8: Gender Disparities**

In some areas, male and female health workers had different roles due to cultural and religious factors. Both genders had equal access to digital payment platforms and mobile phones. Most of the respondents interviewed in this study reported their views on the gender disparities among male and female health workers. Most of them supported the use of e-payment as a payment strategy which gives both genders equal access to digital platforms to receive their payment.

*“All of them are very important in the program as sometimes men can go to areas women cannot go and vice versa. some men of married women may not want them to have phones as they may say they will communicate with people they are not meant to communicate with. Even some that have phones are limited to calling and receiving calls, they will not allow them to do Facebook, WhatsApp and others. Muslim women per se, not Christians. We find out that some of the husbands will not allow Android phones.”* **[Participant 8, Gwagwalada KII]**

*“It is men. Because females sometimes hardly see them with phones like that. If you see them with phones, it is not an Android phone, ordinary Nokia or touch light phones, small phones... From my own side, I think it is not educating, because even the local person in the village who attended primary school must know how to use a phone.”* **[Participant 2, LIO KUJE KII]**

*“Both males and females are always eager to work. The vaccinators and supervisors are mostly females and the community leaders are males because men cannot enter into another person’s house but females can enter and vaccinate the children.”* **[Participant 1, SOKOTO 3 KII]**

*“Yes, they are having access to their phones. The male is more committed to operating their phones. Both male and female have equal access to phones.”* **[Participant 9, OYO KII]**

*“In some exercises, we have to co-opt female and male. For, instance in this FIPV campaign, we don’t have enough vaccinators, or qualified health workers to do the vaccination. So, we are going to use the people system, not house to house system so we don’t have any problem co-opting females and males because of the culture and religion which don’t allow a male to enter any house but a female can enter any house. ”* **[Participant 10, SOKOTO 2 KII]**

**Theme 9: Challenges and Concerns on the Adoption of Digital Payment System**

Some challenges included delays in failed payments, occasional issues with account numbers, and the need for further improvements in the digital payment system. Network issues were mentioned but were not significant barriers. Most of the respondents interviewed in this study reported their views on the challenges facing health workers in carrying out their roles as a vaccinator. Most of the participants supported the use of e-payment as a payment strategy as some challenges such as further improvements in the use of e-payment systems and digital platforms, occasional issues with account numbers, failed payments and also failed transaction processes, and network issues.

*“Transaction failure, we also insist that everybody must have an account number, some whose account details are borrowed denied receiving an alert so we asked them to go and bring a statement of account.”* **[Participant 6, SOKOTO 1 KII]**

*“The challenges are the account details. The participants can't give you what you want and he keeps sending and the organizers keep sending it back to you. It is very stressful on the "L. I" especially when it is a failed transaction. So many names will be sent back and you will have to be calling and calling again for them to reprocess the transaction.”* **[Participant 3, Cross Rivers 3 KII]**

*“You can only find this internet networking majorly around Kuje metropolis. Out of the 10 wards we have in our own area council, I think only 3 are accessible to the network. The remaining 7 don't have a network…. Ok, let me just go and use this money when there is a network... this is the challenge we are facing…”* **[Participant 2, LIO KUJEKII]**

*“The challenge we have is still on this incorrect account details because no matter how much you shout people will still give a wrong number or incomplete digits.”* **[Participant 5, CROSS RIVERS 2 KII]**

*“Apart from delay, failed payment, to be sincere there are no any other challenges…. The other challenge you talked about was that some do not have bank accounts, but you told them and now you told them that all of them must have and about 95% of them have their own bank account. so, this process has ensured that more people have access to bank accounts than before as a result of this.”* **[Participant 4, LIO KWARE LGA]**

**Theme 10: Recommendations**

Respondents suggested the need for ongoing improvements in the digital payment system, such as addressing payment delays and ensuring accurate account information.

Most of the participants interviewed in this study reported their views on the recommendation to improve the use of digital platforms. Most of the respondents supported the use of e-payment as a payment strategy and also recommended an improvement in the use of digital platforms, ensuring accurate and adequate account information of the health workers, and addressing payments and transactions delayed.

*“Policymakers at National, State and Local govt level should find a way to current these challenges so that the vaccinators can be happy and be better motivated to do their work.”* **[Participant 1, Sokoto 3 KII]**

*“ So if it is possible, it is going to be perfect when someone has worked, tomorrow you call him, he will finish his work today. it is just like labourers, when you finish this work, you say okay, take your money. I think it is going to be better that way than for us to say, after finishing your work for ten days, twenty days you will not see your money. So, is there any way that government can improve? ”* **[Participant 2, LIO KUJE KII]**

*“The suggestion I can say is that during this digital, they should be patient and careful in sending the appropriate money to the right allocation at the right time.”* **[Participant 9, Oyo KII]**

*“To the handler of the account details, because most times when you validate, you are sure that what you sent out is correct but when payment is done you begin to see a failed transaction and you begin to wonder so most times people handling it should be more careful when handling the account so that they will not dabber with the numbers maybe in reducing it, increasing it or the numbers disappearing.”* **[Participant 3, Cross Rivers KII]**

*“My recommendation is (1) immunization programme is such that needs motivation for those who will be sent to the field to work … (2) People should be paid not later than a week or at most 2 weeks…they should make us aware as soon as possible so that the Area council can collate, imagine this one now...”* **[Participant 7, AMAC KII]**
