# Supplementary material for: Health workers’ experiences with digital and cash payments during health campaigns in Nigeria: a qualitative study
Source: Front Digit Health. 2026 Oct 23;8:1780807. doi: 10.3389/fdgth.2026.1780807 (PMC13739669; doi:10.3389/fdgth.2026.1780807)
Supplement: Supplementary file 3 [file Datasheet3.docx]

**REPORT FOR QUALITATIVE ANALYSIS RESULTS ON ASSESSMENT OF THE INFLUENCE OF DIGITAL PAYMENT ON HEALTH WORKERS’, MOTIVATION, PERFORMANCE AND RETENTION**

This report presents the analysis output conducted on seven (7) Focus Group Discussion (FGD) transcripts and reveals several common and relevant themes that emerge from the discussions. These themes provide insights into the experiences and perceptions of health workers regarding immunisation campaigns and digital payment systems in Nigeria. The themes identified are as follows: Health workers in the discussions covered various payment methods, including cash and digital transfers, with a notable focus on the transition from cash to digital payments system (DPS). This shift was generally well received, with health workers appreciating the convenience and security it offers. Digital payments were seen as a motivator for improved work performance, encouraging health workers to work harder during immunization campaigns.

Importantly, gender equality prevailed in payment methods, and cultural considerations influenced campaign strategies. Access to digital platforms is facilitated by phone ownership, with Android phones being prevalent among health workers. While digital payments brought benefits, such as security and convenience, challenges, such as delays and account verification, were noted. Suggestions for improvement include accurate account verification and timely alerts. This analysis showed that digital payments positively impacted work motivation and population coverage during campaigns. Using the thematic analysis model of the presentation of key data, this data point shows that eleven (11) themes fit the four (4) study objectives, and they are all listed in the thematic summary table presented below.

**Table 3: Thematic Summary for Analysis Output on FGD Transcripts**

| 1st Objective | Mechanisms through Which Digital Payments Influence Immunization Campaigns in Africa |
| --- | --- |
| **Main Themes** | - **Transition to Digital Payments Systems (1)** - **Adoption of Hybrid Payment Systems for Immunization (2)** |
| 2nd Objective | Influence of Digital Payment on Health Workers’ Satisfaction and Motivation |
| **Main Themes** | - **Positive Influence on Motivation and Work Ethics (3)** - **Positive Influence on Satisfaction (4)** |
| 3rd Objective | Influence of Digital Payment on health workers’ performance and retention |
| **Main Themes** | - **Positive Impact on Work Performance (5)** - **Positive Impact on Immunization Population Coverage (6)** |
| 4th Objective | Influence of gender social norms on digital financial service behaviour |
| **Main Theme** | - **Gender Equality (7)** - **Cultural Inclinations (8)** |
| 5th Objective | Benefits/Risks/Unintended Consequences and Challenges Associated with the Use of Digital Payments for Health Workers |
|  | - **Benefits of Digital Payment (9)** - **Challenges of Digital Payment (10)** - **Suggestions for Improvement (11)** |

**Descriptions of Thematic Summary**

The first research question and objective of this study were answered by the first two themes presented in the thematic summary table below and the implications is that the study has discovered that the act of **“Transiting from conventional payment system to digital payment system”** and **“The adoption of the hybrid payment systems”** were the two mechanisms adopted by the healthcare system decision-makers in implementing the immunization campaigns in Africa in Nigeria. These two themes were coined from the information provided by the discussants in the numerous Focus Group Discussion and the originating quotes were presented in the body of the report after the table presentation.

Similarly, theme three and four were also generated and named to match and answer the second research objective/question as the study discovered that the adoption of digital payment system has **“Motivated the healthcare workforce in such a way that it improved their work ethics”** as well as having **“Positive influence on their job satisfaction”.** The findings from the second objective also linked to the third research questions and objectives where the influence of digital payment system on work performance and workers’ retention was assessed, the study found out that the adoption of digital payment system also had **“Positive impact on the healthcare workforce performance during immunization programme”** and also led to the improvement of **“Immunization population coverage”.**

The study fourth research objective/questions assessed gender and social norms as two key factors that could influence digital financial service behaviour for healthcare programme implementation with special reference to immunization programme and it was discovered that there is **“Gender equality”** in the usage of the digital financial service. However, the study found out that there was **“Cultural inclinations”** on the usage of digital financial services and it bothers on the access to phone ownership as an accessory to making digital financial services accessible to the users. This implies that **“Gender equality and Cultural Inclinations”** were the two thematic expression that answered research objective/question four . Lastly, the study found out that the adoption of digital payment systems had numerous **“Benefits”** as well as specific **“Challenges”** that is limiting its wholly adoption as the sole payment system for healthcare workers remuneration and honorarium during immunization programme. In hindsight, the discussants engaged in the focus group discussion provided some **“Suggestions”** that they perceived to have the potential for the improvement of the digital payments, which makes the third thematic expressions that answered the fifth research questions/objectives.

**Theme 1**

**Theme 2**

**Research**

**Objective 1**

Research Question 1

**Theme 9**

**Theme 10**

**Theme 11**

**Theme 7**

**Theme 8**

**Theme 5**

**Theme 6**

**Theme 3**

**Theme 4**

**Diagrammatic Representation of Thematic Summary Table**

**Research**

**Objective 5**

**Research**

**Objective 3**

**Research**

**Objective 2**

**Research**

**Objective 4**

Research Question 5

Research Question 4

Research Question 3

Research Question 2

**Thematic Map**

Benefits of Digital Payment

Gender Equality

Positive Impact on	Work Performance

TAP

GO

SO

Transition to Digital Payment System

Positive influence on Satisfaction

Challenges of Digital Payment

Positive Influence on Motivation and Work Ethics

TE

Cultural Inclinations

Positive Impact on Immunization Population Coverage

Adoption of Hybrid PS for Immunization

Suggestions for Improvement

**Key**

TAP: Thematic Analysis Map

GO: General Objective

SO: Specific Objectives

TE: Thematic Expressions

**Descriptive Analysis Supporting Evidence with Quotes**

**Theme 1: Transition to Digital Payments**

Discussants express positive sentiments about the transition from cash to digital payment citing benefits such as increased convenience reduced stress of receiving payment under unfavourable conditions while staying in a comfortable space to receive payment, getting paid for the work done and improved work on the field, easy payment and accessibility to the money being paid which consequently motivates them to work. Discussants also talked about the security measures digital payment has incorporated in compromising cash payments and reducing or eliminating the issue of ghost workers.

“*The digital is preferable in the campaign performance. Digital has been reflecting positively on the work field*” – [**Discussant 1**, **AMAC FGD 1]**

“*People are willing to work. People are really willing to work because they have seen that the payment modality has improved.*” - [**Discussant 6, Cross River State FGD 1]**

“*As for me, E-payment is better because if we should compare the situation we are in now, it is very risky for someone to carry money all about, with that, E-payment is better. Also, cash payment can be compromised, there may be ghost workers as well, by going to the cashier and presenting the necessary documents for the payment without being part of the Job. Lastly, E-payment is very important if you compare with recent work completed five immediately, we completed the work they paid us in time*” [**Discussant 11, Cross River State FGD 1]**

“*In this issue of digital payment, it is very important compared to the cash payment system that takes a longer time before we receive the money but now within two weeks, we receive the money that is the reason why we prefer this digital payment.*” - [**Discussant 7, Sokoto FGD 2]**

“*The benefit there is that at least when I work, I will receive my money anyhow. I will not be expecting that oh, this person is coming to pay me and I will fill the slip and I won't see it as my own*” - [**Discussant 2, Cross River State FGD 2]**

“*It is only few who are sincere, if some are sent to give two thousand to give to a sister, some of them will give one thousand and corner the one thousand. so this one that is digital we want it and even our governor was digital so whatever you receive in your account, you will know that that is for you. so we accepted that one*.” - - [**Discussant 3, Cross River State FGD 2]**

***“****It reduces the stress of having to queue and get paid. but this one even if it is POS, I will just withdraw my money***”** - [**Discussant 7, Cross River State FGD 2]**

“*There is a benefit. for the transfer, normally, when you are working, there is a particular card you are going to fill, in that contains certain information like your daily payment, if you calculate all your daily work payments you will receive the money if it is through the bank but if it is table payment, there are times we don't get all the money*” **– [Discussant 2, Kuje FGD 2]**

“*There is, maybe you are not around, or you are sick, or you have an excuse. you don't have to drop whatever you are doing to come to where they are giving the cash. you will receive your alert from wherever you are. So I think that is the benefit*”- **[Discussant 3, Kuje FGD 2]**

“*The benefit is maybe you are far away; you don't have to travel down to collect your money. I could just receive an alert and that is one of the advantages of the bank payment. For example, if they say we should go and collect the money, we might not be opportune to go there. if they are paying through the bank, you will just receive your alert, there is no stress when you want to collect your money* ” **-[Discussant 5, Kuje FGD 1]**

“*Yes. It is very simple to pay by bank transfer instead of cash. It has encouraged me to work better. We are happier if they send through bank account than through cash*” - **[Discussant 2, Sokoto FGD 2]**

**Theme 2: The Adoption of Hybrid Payment Systems**

The discussants collectively provided their expressive opinions on the type of payment system that they are familiar with within the healthcare system, which showed that there is a merging of both cash and digital payments as the majority of the discussants shared their experiences on the transition, usage of the old system as a way of covering the shortcomings of the digital payment systems and preferences for digital transfer. The justification for the preferences was based on the belief that it increases the speed at which money is allocated for payment, and helps to receive payment from far proximity irrespective of the location where the payment was initiated. However, some of the discussants prefer cash payment to digital payment because it reduces fraudulent activities with the assurance that only legitimate workers who were available for work get paid and avoidance of banking problems; avoidance of delayed bank alerts and reversed payments. The following quotes were extracted from the discussants’ direct responses:

*“For me, I would prefer the payment in cash over the bank account payment because at times after the money has been sent to the workers' bank account, we don't see the alert*” – **[Discussant 1 AMAC FGD]**

“*Before we were receiving the cash payment and now, we are receiving it digitally. I like the digital payment because there are some days someone may not be present at the location of the payment or probably have been transferred to another area, I am confident that I will receive my payment digitally*”- **[Discussant 4 Sokoto FGD 2]**

“*These types of payments, either cash or digital. As for me cash payment is the most preferable because when it is payment time, you go with your card they will see you and give you your money but if it is E-payment, at times they usually do ghost workers because they don’t see the person to pay. But as for cash payment, you will see my picture, my name and all my details in the record and I will be the one to collect the money*”- **[Discussant 10 Sokoto FGD 2]**

“*When I joined, cash payment was used then to pay for transportation but for the immunization campaign, I was paid by transfer. When it comes to programmes like polio, they are being paid in two ways. The first is for transportation and the second, is payment for the immunization campaign which is both digital. I prefer being paid by transfer for the transportation and the reason for this preference is because when we were being paid by cash, the money allocated then was lower compared to when we were paid by transfer”*- **[Discussant 1, Ibadan Southwest FGD 1]**

“*I prefer cash. Most times they collect our account details and we don't receive the money. like the last measles that we did, we have not received the money till today*” – **[DISCUSSANT 2, Kuje FGD 1]**

“*I used to collect cash before and now they pay us through transfer. Before we receive our money, we wait for 3 months but now for the work we finished last week, they pay us early through transfer*” **[Discussant 6, Sokoto FGD** **2]**

**Theme 3: Motivation and Work Ethic**

Health workers describe how digital payments motivate them to work harder and perform better in their roles. The assurance of prompt payment encourages them to be more diligent ensuring that they reach the depths of the community during immunisation programs.

“*Yes, this E-payment motivates health personnel because they know that as soon as you present your account details and your account doesn’t have an issue, you will receive the alert immediately therefore it motivates you because you know when you work you will be paid*.” - [Discussant **10, Sokoto FGD 2]**

“I*t has improved people’s system of work. They expect money, and it comes on time. It made people even more agile towards work*” - [Discussant **12, Ibadan Southwest FGD]**

“*It makes us more excited to work hard and cover up in time because we know an alert will come when we finish the work. Knowing that you are going to be paid in a short time will make you more excited to go to the field and work*.” - [Discussant **4, Sokoto FGD 1]**

**Theme 4: Positive Influence on Satisfaction**

The findings also showed that the transition from old methods of payment to digital payment contributed to healthcare workers' job satisfaction as they identified the new payment method as an innovation that would solve the inherent problems with the advantages of digital payments over traditional cash disbursements in various contexts. They were satisfied with their job and payment because digital payment provides easy accessibility to their payment, timely compensation and eliminates excuses for delayed payments. Their excitement about digital payment also hinges on its advantages including the promotion of responsible spending, saving time, reducing the stress that would be wasted staying in a queue, and encouraging savings.

“*Easy accessibility of the money been paid to you and there is that motivation. you are sure of getting that satisfaction for what you have done. With that, you will be forced to take another campaign when it comes. you will work and get paid, not when you will work and somebody will be giving you some excuses about the pay. So, I feel the digital payments is beneficial for those that have worked, they really achieved what they have worked for and it is easy to get it without any stress with no obstacles apart from some charges that are been imposed by the banks”* - [**Discussant 5, Cross River State FGD 2]**

“*Giving us through a bank account is more exciting and satisfactory than giving us through hand because we save our money till, we need it to buy something. Giving us cash can make us spend it anyhow*” - [**Discussant 2, Sokoto FGD 1]**

“*It makes the workers happy and it saves stress and time of queuing to collect money by hand, it also motivates and helps to plan savings. It makes those that are finding it hard to secure a personal bank account open their own bank account in other to avoid not paid while the rest are*” – [**Discussant 6, AMAC FGD]**

“*They were paid through the account.* *I want to… I want to… I’m very happy about this coming up now. Because I have been a victim once or twice, after the work, no payment. The last OBR, I didn’t see the money*. *Last year! I think early last year. I didn’t see the payment as a supervisor. I didn’t see the money. I was supervising the campaign. I’m not the only person, because, others too do complain in past campaigns. Sometimes some people will receive the money, others will not. But, the same account details. The same account details. They keep saying, there is a problem, resend, (echoes) resend the account, keep resending, keep resending”* - [**Discussant 2, AMAC FGD]**

“*We are satisfied with the use of digital but there should be an improvement. Such as no transaction failure, discussants should receive their money in time.*” - [**Discussant 11, Ibadan Southwest FGD]**

**Theme 5: Impact on Work Performance**

Health workers in several transcripts highlight how digital payments have positively impacted their work performance. Discussants highlight the impact of timely digital payments that motivate them to work harder by encouraging health workers to work efficiently and effectively as well as improving population coverage during immunization campaigns such as increasing health workers' participation in the campaign to mobilize more people for immunization.

“*People are willing to work. People are really willing to work because they have seen that the payment modality has improved*” [**Discussant 6, Cross River State FGD 1]**

“*The overall impact of E-payment is to assist. Most of the children's fathers agree to vaccinate their children because they are very appreciative to welcome the health workers when they come to immunize their children and that is the reason we the health workers are trying to enhance the system of immunization in order to have full direct immunization coverage*” **[Discussant 9, Sokoto FGD 2]**

“*Yes, this E-payment motivates health personnel because they know that as soon as you present your account details and your account doesn’t have an issue, you will receive the alert immediately therefore it motivates you because you know when you work you will be paid*. *Like now, we worked on the net campaign and immediately we completed and submitted the necessary documents on the net campaign about 2weeks later everybody kept saying they had gotten alert, therefore it motivated and ginger.*” - **[Discussant 10, Sokoto FGD 2]**

“*If I may say, the overall is motivating the discussants because before it took a long time, about 3 weeks to 1 month before being paid on the other hand, E-payment is very easy to get your payment in time and the payment time for the workers is very motivating. The E-payment helps us to track the actual personnel during the implementation of the process which is why the E-payment system is very impactful. The E-payment motivates the health workers because when your bank detail is inputted on the platform and you are also confident that your account detail is correct, when they pay you will definitely receive your own alert and that is the reason it motivates them to work hard*” - **[Discussant 11, Sokoto FGD 2]**

“*Yes, people are turning up, no dropouts amongst the health workers. The volunteers are also more interested in the programmes*.” - **[Discussant 10, Ibadan Southwest FGD]**

**Theme 6: Impact on Population Coverage**

The discussants opined that the use of digital payments positively affects the rate at which people receive them and extends to wider population coverage during immunization campaigns. Because of the guarantee that their payment will come as at when due without going to queue and ask for it at the centre. The immunization campaign will be more successful as the vaccinators are motivated to work longer hours, leading to better coverage.

“*I will say yes and no. Because, initially, people see us as people who are collecting so much money and as we go out to meet people, some of them will receive us wholeheartedly. Some will say, whether after all, you people do the work or not, they will still pay you. Meanwhile, it’s not like that, like, the health workers, we do the work first, if payment comes if it doesn’t come, we are already in the system. So, like this last payment that we were paid and the money got straight into the various persons' account, they were happy, happy to even do the work again*.” - [**Discussant 1, Cross River State FGD 1]**

“*People are willing to work. People are really willing to work because they have seen that the payment modality has improved*” - [**Discussant 6, Cross River State FGD 1]**

“*Yes, this E-payment motivates health personnel because they know that as soon as you present your account details and your account doesn’t have an issue, you will receive the alert immediately therefore it motivates you because you know when you work you will be paid*. *Like now, we worked on the net campaign and immediately we completed and submitted the necessary documents on the net campaign about 2 weeks later everybody kept saying they had gotten alert, therefore it motivates and ginger.*” [**Discussant 10, Sokoto FGD 2]**

“*The payment either cash or transfer being given to the discussants at the right time increases the morale of the discussants which makes them come to work quickly and this will curb the increase of polio cases. The number of non-compliance has drastically reduced over the years. So, they are cooperating now*” - [**Discussant 1, Ibadan Southwest FGD]**

**Theme 7: Gender Equality**

The discussants consistently mention that gender does not play a role in determining payment amounts or methods, and there is no significant gender-based difference in phone ownership. Access to digital payment platforms is facilitated by either owning a phone or opening bank accounts therefore, discussants mention that both genders have equal access to owning a phone and payment is received with a bank account that is active with correct details. The discussants further mentioned that both male and female health workers receive equal compensation for their work, irrespective of the payment method though there are more female health workers than male health workers.

“*They have equal access because in E-payment it does not matter whether you are a male or female in as much your account is active and correct you would be paid*.” – [**Discussant 10, Sokoto FGD 2]**

“*Male and female have equal access to digital payment, the amount they give to men is same as what they give to women, no disparity*.” [**Discussant 10, Ibadan Southwest FGD]**

“*It makes those that are finding it hard to secure a personal bank account open their own bank account in other to avoid not paid while the rest are*” - [**Discussant 1, AMAC FGD]**

“*Initially, (one house) for those who are willing to work but don’t have an account number, they normally bring their relation account number name. But in our own ward here, my madam (Name withheld) won’t accept, because she wouldn’t want any complaint. So, you must have your own account*” - [**Discussant 4, Cross River State FGD 1]**

“*Sir, we are in a digital world, 99.9% have phones because of the way things are going globally now. it will be hard for you to see someone without a phone, even though it is this normal torchlight phone*.” - [**Discussant 5, Kuje FGD]**

**Theme 8: Cultural Considerations**

Cultural beliefs and practices related to gender roles and household visits during immunization campaigns are discussed. Few discussants made responses to the cultural considerations that influence the way health workers conduct their activities. The few discussants were made up of the two fractions. The first fraction of discussants mentioned that there were no cultural beliefs or practices related to gender roles during payment activities while the other fraction mentioned that there is a cultural practice for gender roles during cash payments and household visits during the campaign.

“*That was what I said; they used to form two lines, one for females and one for males because of our culture. They used to call number, when they take 10 females, they will take 10 males too but now with the digital payment they pay equally*” - [**Discussant 11, Sokoto FGD 2]**

“*There is no religion or cultural beliefs to support unequal access to digital payment platforms*.” - [**Discussant 10, Ibadan Southwest FGD]**

“*They take women for OBR and NIPDS because of our religion and culture. The man stays outside so if you have any problem, you come and explain to him*” - [**Discussant 6, Sokoto FGD 1]**

“*According to Muslim tradition, you cannot allow males to enter the house. You can only allow females to enter and talk with their parents. If they are not interested in it, they will tell them to meet with the community leader*” - [**Discussant 2, Sokoto FGD 1]**

**Theme 9: Benefits of Digital Payments**

Health workers share numerous advantages of digital payments, including increased security, convenience, and the ability to save money for future needs. Discussants view digital payments as a positive change from cash payments with the benefits of prompt and easy payment, securing and safeguarding their money from misappropriation and theft, which as a result motivates health workers to save their money for future purposes.

“*You get your money, the exact amount. No addition, no subtraction. No deduction of any kind. For all the categories, you know, all the ward vocals collect differently. But, it’s the same for all the ward vocals, the supervisors get the same amount across the LGAs, the vaccinators get the same amount all over, the Red Codgers, the town announcers, the community leaders, they get exactly what is scheduled for them across boards*.” [**Discussant 3, Cross River State** **1]**

“*There is a benefit as you have said because before it took a long time before we received payment but now with E-payment within 2 weeks to 3 weeks everybody receives their payment. It is very good*.” - .” [**Discussant 1, Sokoto FGD 2]**

“*It is very good because before you don’t get paid until three to four months but now it is between one to two weeks that one receives their alert*” [**Discussant 2, Sokoto FGD 2]**

“*The benefit of this E-payment is very good because of the security issues. Assuming you go another LGA to receive your payment while coming you may get robbed but now, they paid into the account*” [**Discussant 7, Sokoto FGD 2]**

“*It is very fast as I said earlier on, conducive because people receive their money faster and easier and there won’t be any inconveniences. In terms of transparency, I will be particular about transportation. When they were paying cash, there was no transparency, it was just what was being allocated to them that they got. I could remember, that the amount paid to the supervisor was more than the amount paid to mobilizers, but with this digital, it was direct to their accounts, and they have an equal share of the money. There is transparency in digital payment. It has reduced the incidence of theft.*” [Discussant 12, **Ibadan Southwest FGD]**

“*The benefit is that the payment that is for you is what you will get. No one will deprive you because once it has entered your account, you own it. After all, if it enters somebody's account, the person can deprive you of owning it. So, we like the digital payment*” - **CROSS RIVER 6**

“*It reduces the stress of having to queue and get paid. but this one even if it is POS I will just withdraw my money*.” - **CROSS RIVER 7**

“*The benefit is maybe you are far away, you don't have to travel down to collect your money. I could just receive the alert and that is one of the advantages of the bank payment. For example, as we are here, if they say we should go and collect the money, we might not be opportune to go there. if they are paying through the bank, you will just receive your alert, there is no stress when you want to collect your money*.” - **Kuje 5**

“*You have to waste transport money to collect your payment in cash but now you can be at home and receive the alert. Someone can rob you of the money or you even lose it during the cash payment. But in your account, you can remove it through POS*” - **Sokoto 4**

**Theme 10: Challenges with Digital Payments**

The majority of the discussants reported the challenges related to digital payments which include delays in receiving the alert for payment, network issues, and the need for proper account verification, the potential for incorrect account numbers and issues with banks are also mentioned as the challenges faced. In some situations, only a few of the immunization workers receive alerts. Another challenge identified is that the problem is not rectified, some of the discussants stated they logged their complaints on delayed payment but no one attended to it.

*“Some confusion payment issues just that the delay is quite alarming especially for the people in the remote area and due to impatience of these same people, they tend to give up easily on the rectification of their money. Also, most of these workers are people from the community and most of these people don't have a personal bank account so, they can share the same account with their friends or families when it's time for payment, WHO will end up just sending one money probably because they are very careful against a fraudulent act that is, sending money to the same account number but different names*” **[Discussant 3, AMAC FGD]**

“*The only challenge is the failed payment as my colleague has mentioned, there are some failed payments about two years now that are yet to be rectified and if they are going to rectify that we would be very happy*.” **[Discussant 3, Sokoto FGD 2]**

“*To me, the challenge we are facing is the borrowing of account and some are neglecting to go and open their account that’s why we have the issue of failed payment, some are not failed payment but hidden payment*.” **[Discussant 11, Sokoto FGD 2]**

“*Inconsistency in the collation of account number. there used to be mistakes. Same question, same answer. The inconsistency of the device or oversight of the device to collate the account number to correct.*” **[Discussant 2, Cross River FGD 2]**

“*You will still receive your money, at least the aim is achieved, but now we don't have anybody to complain to, even if you complain, you will be told to go to your bank*.” **[Discussant 3,** **Kuje FGD]**

**Theme 11: Suggestions for Improvement**

The discussants offer suggestions to enhance digital payment systems, such as ensuring accurate account verification, timely distribution of payment alerts, and continued support in opening bank accounts. Some of them express the need for improved logistics during immunization campaigns.

“*My suggestion is two; firstly, the organization should pay in time, secondly, the focal person should verify the account before you submit it*.” **[Discussant 4, Sokoto FGD 2]**

“*Once trusted personnel is put in charge and once well-arranged account numbers are collated, the person cannot manipulate but once someone who loves money is put in charge, the person will manipulate the payment*.” - **[Discussant 2, Ibadan Southwest FGD]**

“*If an application that can collate and validate each person’s account is being built, for example, the COMCARE if it can be improved on, sent to supervisors to crosscheck before they make the payments, this can help improve the payment and facilitate it*.” **[Discussant 1, Ibadan Southwest FGD]**

“*The money should be paid. To implement it. Those banks that are supposed to pay the beneficiary, I feel if they are involved maybe especially the MD of the bank telling them to release the cash in less than 24 hours. So, I feel bankers should be involved in the process of this payment. So, the beneficiaries can get the money immediately the organization pays in their checks*.” - **[Discussant 5, Cross River FGD1]**

“*Not a question but more like a suggestion. sir, when this issue happens and I get to meet someone like you, I will be fair, maybe there should be more of you in a program like this that we can channel our problems to or more like e-mail stuff or phone number. anything that we can send our complaint to. not like the one when we will meet people and they will say they don't have any of them. how sure am I that if I give you a message, that the message is going to be passed across to them? So, I prefer there should be more like you. it might not be physically, but I guess phone number, e-mail*.” **[Discussant 3, Kuje FGD]**

“*Once your friend has been paid and you have not seen yours, you can call your focal person to tell him so he can verify your bank account. Once the bank pays, they should send the alert to all health workers without delaying for 2 or 3 days*” **[Discussant 2, Sokoto FGD 1]**
